# Pharmacokinetic, Safety, and Tolerability Evaluations of Gepotidacin (GSK2140944) in Healthy Japanese Participants

**DOI:** 10.1002/cpdd.1192

**Published:** 2022-12-05

**Authors:** Aline Barth, Mohammad Hossain, Caroline R. Perry, Annette S. Gross, Hirofumi Ogura, Shaila Shabbir, Sebin Thomas, Etienne F. Dumont, Darin B. Brimhall, Meenakshi Srinivasan, Brandon Swift

**Affiliations:** ^1^ GSK Collegeville Pennsylvania USA; ^2^ Present affiliation: Global Blood Therapeutics San Francisco California USA; ^3^ Present affiliation: Servier Pharmaceuticals Boston Massachusetts USA; ^4^ GSK R&D Sydney AU; ^5^ GSK Tokyo Japan; ^6^ GSK Stevenage UK; ^7^ Present affiliation: Boston Pharmaceuticals Cambridge Massachusetts USA; ^8^ PPD Las Vegas Nevada USA; ^9^ GSK Durham North Carolina USA

**Keywords:** food effect, gepotidacin, Japanese, pharmacokinetics, relative bioavailability

## Abstract

Gepotidacin is a novel, bactericidal, first‐in‐class triazaacenaphthylene antibiotic in late‐phase development for uncomplicated urinary tract infection and uncomplicated urogenital gonorrhea. Two clinical studies were conducted to assess the pharmacokinetics (PK) and interethnic comparisons of oral gepotidacin (free‐base and to‐be‐marketed mesylate formulations) administered as single doses ranging from 1500 to 3000 mg in fed and fasted states, and as 2 × 3000‐mg doses given 12 hours apart under fed conditions in healthy participants of Japanese ancestry. Dose proportionality was observed in plasma exposures, and comparable area under the concentration‐time curve (AUC) and maximum concentration were observed in fed and fasted states. Interethnic comparisons for Japanese versus non‐Japanese participant data showed slightly higher plasma maximum concentration (7%‐30%) yet similar plasma AUCs; slightly lower urine AUCs (11%‐18%) were observed. The slightly higher plasma exposures in healthy Japanese versus White participants in the same study were attributed to lower mean body weights (64 kg versus ≈80 kg). Adverse events were primarily gastrointestinal, and when administered with food, gastrointestinal tolerability was improved. Overall, the gepotidacin PK and safety‐risk profiles in healthy Japanese support potential evaluation of the global clinical doses in future studies.

Gepotidacin is a novel, bactericidal, first‐in‐class triazaacenaphthylene antibiotic that inhibits bacterial DNA replication by a distinct mechanism of action,^1^, ^2^ which confers activity against most strains of target pathogens, such as *Escherichia coli*, *Staphylococcus saprophyticus*, and *Neisseria gonorrhoeae*, including those resistant to current antibiotics.^3^, ^4^, ^5^, ^6^, ^7^ Phase 2 clinical studies conducted in acute bacterial skin and skin structure infections, uncomplicated urogenital gonorrhea, and uncomplicated urinary tract infection (uUTI) substantiated the antimicrobial activity and safety profile of gepotidacin for further evaluation.^4^, ^8^, ^9^, ^10^ Thus, phase 3 clinical studies are under way to assess gepotidacin in uncomplicated urogenital gonorrhea when administered as 2 × 3000‐mg oral doses separated by 10‐12 hours (NCT04010539)^11^ and in uUTI as a 1500‐mg oral dose taken twice daily for 5 days (NCT04020341 and NCT04187144).^12^

Gepotidacin pharmacokinetic (PK) and safety evaluations have been predominantly conducted in healthy Western, predominantly White, participants.^13^, ^14^, ^15^, ^16^, ^17^, ^18^ As interethnic differences in drug PK and safety have been reported for other drugs,^19^, ^20^ the objective of the studies presented herein was characterization of the gepotidacin PK and safety profiles in healthy participants of Japanese ancestry.

Gepotidacin elimination and metabolism have been evaluated in healthy participants administered single intravenous and oral doses of 14C‐gepotidacin.^13^ Following oral administration (capsule formulation), ≈50% of the dose was absorbed. Gepotidacin was eliminated mostly unchanged in urine accounting for ≈43% and 20% of the dose following intravenous and oral administration, respectively. Elimination via metabolism (urine plus feces) accounted for a total of ≈19% and 13% of the dose by the intravenous and oral routes, respectively. Cytochrome P450 (CYP) 3A4 was found to be the major enzyme responsible for the oxidative biotransformation of gepotidacin.^13^

A number of studies have reported that the average activity of CYP3A4 is similar,^21^, ^22^, ^23^, ^24^, ^25^ or slightly lower,^26^, ^27^, ^28^ in populations of East Asian ancestry from Japan and China relative to populations of European ancestry. These observations in vivo are supported by in vitro experiments indicating that CYP3A4 expression and activity are similar in liver samples from Japanese and White participants.^29^ Tateishi et al^26^ concluded that any difference in CYP3A4 activity between Japanese and European populations is small and of limited clinical significance. Therefore, significant interethnic PK differences due to CYP3A4‐mediated metabolism of gepotidacin are not anticipated.

With the major route of elimination of gepotidacin being renal clearance via glomerular filtration and active tubular secretion, the contribution of drug transporters to renal elimination has been investigated. Although gepotidacin is a substrate of multidrug and toxin extrusion (MATE) 1 and MATE2‐K, these transporters are considered to have a minimal impact on gepotidacin exposure in vivo. Surprisingly, gepotidacin was not transported by basolateral renal solute carrier transporters including organic cation transporter 2/3 and organic anion transporters 1/2/3. Furthermore, preclinical studies have ruled out P‐glycoprotein and breast cancer resistance protein as making notable contributions to gepotidacin transport (NCT04493931/manuscript in preparation). For many drugs principally eliminated via the urine, major interethnic differences in PK have not been reported.^19^

Two gepotidacin PK studies in participants of Japanese ancestry have been performed to characterize the PK and safety profiles of oral gepotidacin in this population. These studies were conducted to support expansion of gepotidacin clinical development to Japan. Following the initial PK study in healthy Japanese (BTZ117351; NCT02853435), there was a change in tablet formulation, and to meet requirements to assess the PK and effect of food on PK of the formulation to be used in patients after regulatory approval, a second PK study (213678; NCT04493931) was conducted. This second study also included a within‐study interethnic comparison of gepotidacin PK and safety. Results from these 2 clinical studies conducted in an ethnic Japanese population are presented herein.

## Methods

### Study Design

#### Ascending‐Dose Study

This study (BTZ117351, NCT02853435) was conducted at Anaheim Clinical Trials, LLC (Anaheim, California), received approval from Aspire IRB (Santee, California), and was conducted in compliance with the principles of the Declaration of Helsinki. All participants provided written informed consent for study participation before initiation of the study. Part 1 of the study has recently been reported elsewhere.^18^ Briefly, it was a 3‐period, crossover study assessing the relative bioavailability of a single 1500‐mg dose of gepotidacin in 2 free‐base tablet formulations compared with the mesylate salt reference capsule formulation. Parts 2 and 3 of the original study are reported herein and have been renumbered to parts 1 and 2, respectively. The objectives were to evaluate the PK, dose proportionality, safety, and tolerability of single, ascending oral doses of gepotidacin in healthy participants of Japanese ancestry under both fasted and fed conditions.

**Figure 1:**
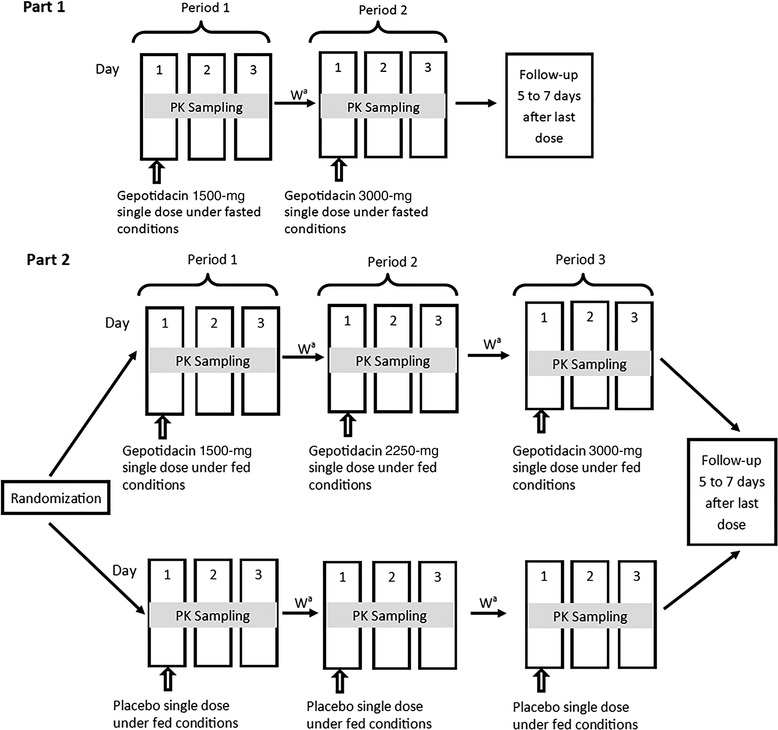
Design of the ascending‐dose study. Gepotidacin was administered as the free‐base formulation. ^a^There was a washout of at least 3 days between each study drug administration. PK, pharmacokinetic; W, washout.

Part 1 of the study was a 2‐period, fixed‐sequence evaluation of ascending, single oral doses of gepotidacin (free‐base formulation) under fasted conditions in healthy Japanese participants (Figure 1). A single 1500‐mg (2 × 750‐mg tablets) dose of gepotidacin was administered in period 1, followed by a washout of at least 3 days, which represents at least 6 half‐lives (t_1/2_; 11.5 hours),^17^ and then a single 3000‐mg (4 × 750‐mg tablets) dose was administered in period 2. With the exception of water, food or drink was not allowed for 10 hours before dosing.

Part 2 of the study was a 3‐period, randomized, double‐blind, placebo‐controlled, fixed‐sequence evaluation of ascending, single oral doses of gepotidacin (free‐base formulation) under fed conditions in healthy Japanese participants (Figure 1). Participants were stratified by sex and randomly assigned to receive gepotidacin as 1500‐mg (2 × 750‐mg tablets), 2250‐mg (3 × 750‐mg tablets), and 3000‐mg (4 × 750‐mg tablets) single doses in periods 1, 2, and 3, respectively, or to receive placebo for all 3 periods. There was a washout of at least 3 days between doses. All doses were administered within 30 minutes after completion of a standard Japanese meal. For each dose level, a sentinel group of up to 4 participants received the study drug to allow assessment of safety and tolerability before the remaining participants received the same dose level.

For both study parts, participants were confined to the study site for the duration of the study until all scheduled assessments were completed, and then returned for a follow‐up visit ≈5‐7 days after administration of the last dose.

#### To‐Be‐Marketed Formulation Study

This study (213678, NCT04493931) was conducted at the PPD Clinical Research Unit (Las Vegas, Nevada), was approved by the Advarra Institutional Review Board (Columbia, Maryland), and was conducted in compliance with the principles of the Declaration of Helsinki. All participants provided written informed consent for study participation before initiation of the study.

The objectives were to evaluate the PK, food effect, safety, and tolerability of the to‐be‐marketed gepotidacin formulation in healthy participants of Japanese ancestry, including interethnic PK data comparisons to healthy Western participants. The full study design included 4 cohorts; 1 comprised healthy Japanese participants to assess the described key objectives and 3 comprised healthy Western participants that allowed within‐study comparisons across Japanese and Western (non‐Japanese) participants (these cohorts were also designed to assess drug‐drug interactions).

**Figure 2:**
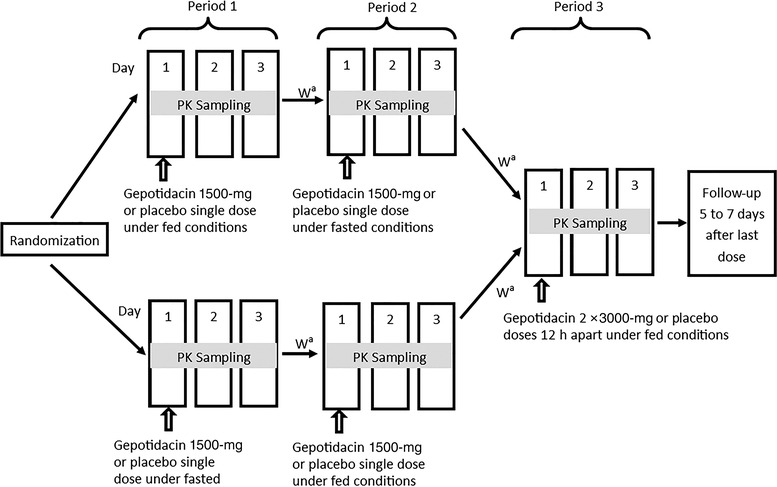
Design of the to‐be‐marketed formulation study. Gepotidacin was administered as the mesylate formulation. ^a^There was a washout of at least 3 days between each treatment. PK, pharmacokinetic; W, washout.

This was a double‐blind, placebo‐controlled, randomized study to evaluate the phase 3 to‐be‐marketed mesylate formulation of gepotidacin or placebo (10 active, 2 placebo) administered as 1500‐mg (2 × 750‐mg tablets) single doses under fed and fasted conditions (periods 1 and 2, respectively) in a randomized sequence, and to assess 2 × 3000‐mg doses given 12 hours apart under fed conditions (period 3) in healthy Japanese participants (Figure 2). The gepotidacin dose evaluated in period 3 represents the maximum total daily dose intended for clinical use (ie, 6000 mg with food) and is the same dose undergoing phase 3 evaluation for uncomplicated urogenital gonorrhea (ie, 2 × 3000‐mg oral doses separated by 10‐12 hours [NCT04010539]). Participants were randomly assigned to receive either gepotidacin or placebo. There was a washout of at least 3 days between doses.

For the fed condition in periods 1 and 2, study drug administration occurred within 5 minutes after completion of a standard Japanese meal. A standard Japanese predose meal was composed of 11% (100 kcal) protein, 40% (348 kcal) fat, and 49% (427 kcal) carbohydrate. For the fasted condition, only water was allowed for 8 hours before dosing. In period 3, both doses of the study drug were administered under fed conditions, which consisted of a standard Japanese meal in the morning and a second meal in the evening that was similar in calorie and macronutrient content to the breakfast meal; all participants were served the same meals during in‐house clinical conduct. In all 3 periods, no food or water was allowed for 2 hours after dosing; water was permitted if required per the investigator for treatment of an adverse event (AE).

Participants were confined to the study site for the duration of the study until all scheduled assessments were completed, and then returned for a follow‐up visit ≈5–7 days after administration of the last dose.

In the 3 Western (non‐Japanese) cohorts, PK assessments were primarily performed to assess drug‐drug interactions (to be reported in a separate manuscript in preparation); data from the gepotidacin‐only arms were extracted and used herein for interethnic PK comparisons. The Western participants underwent the same PK procedures (eg, study site confinement, dosing procedures, and PK sample collections) as the Japanese participants with the exception of Western dietary meals; thus, allowing direct interethnic comparisons. For interethnic comparison of a single 1500‐mg dose, gepotidacin was administered alone. For interethnic comparison of 2 × 3000‐mg doses given 12 hours apart, data were as follows: dose 1 was gepotidacin administered alone, and dose 2 was gepotidacin administered alone (Japanese participants) or gepotidacin coadministered with digoxin and midazolam (Western participants, derived from other cohorts of the overall study design to assess gepotidacin as a potential drug‐drug interaction perpetrator, not victim). The Western participants data are presented for All Comers (defined as all participants, with the exception of Japanese) and a White‐only subgroup. The White‐only group consisted of participants who self‐identified as White/European/Caucasian ancestry.

### Study Participants

Both studies were conducted in the United States in healthy adult male and female participants of Japanese ancestry. All participants were required to be Japanese citizens with a Japanese passport, have 2 Japanese parents and 4 Japanese grandparents (all Japanese citizens), and had lived outside of Japan for <10 years to ensure that the healthy Japanese participants in the United States were representative of Japanese in Japan. All enrolled participants were healthy as determined by the investigator based on a detailed medical history, clinical laboratory results (serum chemistry, hematology, urinalysis, and serology), vital sign measurements, 12‐lead electrocardiogram (ECG) results, and physical examination findings.

#### Ascending‐Dose Study

In part 1, all 10 enrolled participants of Japanese ancestry completed the study. Participants were a mean (range) age of 55.6 (44‐64) years, with more female (n = 8) than male (n = 2) participants, and had a mean (range) body weight of 60.0 (50.1‐73.7) kg (Table 1). In part 2, 11 of the 12 enrolled participants (91.7%) of Japanese ancestry completed the study; 1 male participant withdrew consent after receiving 2 single doses of gepotidacin (no AEs were reported for this participant). Participants in part 2 were younger than the participants in part 1, with a mean (range) age of 37.8 (28‐56) years; an equal number of male and female participants enrolled, and had a mean (range) body weight of 64.2 (50.1‐79.8) kg (Table 1).

**Table 1:** Japanese Participant Demographics and Baseline Characteristics

	Ascending‐Dose Study^a^ Part 1, Fasted (N = 10)	Ascending‐Dose Study^a^ Part 2, Fed (N = 12)	To‐Be‐Marketed Formulation Study^b^ (N = 14)
Age, y	55.6 (6.43)	37.8 (7.93)	37.9 (5.97)
Sex, n (%)			
Female	8 (80)	6 (50)	6 (43)
Male	2 (20)	6 (50)	8 (57)
BMI (kg/m^2^)	22.9 (2.72)	22.8 (3.41)	23.6 (2.54)
Height (cm)	162 (5.92)	168 (8.78)	166 (6.60)
Weight (kg)	60.0 (7.93)	64.2 (10.9)	65.0 (9.49)
eGFR^c^ (mL/min/1.73 m^2^)	…	…	98.9 (15.5)
CLcr^c^ (mL/min)	…	…	110 (15.5)

BMI, body mass index; CLcr, creatinine clearance; eGFR, estimated glomerular filtration rate; SD, standard deviation.

Data are presented as arithmetic mean (SD) unless noted otherwise.

^a^Free base formulation.

^b^Mesylate formulation.

^c^eGFR was derived by the Modification of Diet in Renal Disease formula and estimated CLcr was derived by Cockcroft Gault equation.

#### To‐Be‐Marketed Formulation Study

All 14 enrolled participants of Japanese ancestry completed the study. Participants were a mean (range) age of 37.9 (30‐48) years, with a higher male (n = 8) than female (n = 6) enrollment, and had a mean (range) body weight of 65.0 (47.1‐80.5) kg (Table 1).

For interethnic PK comparisons at the 1500‐mg dose, data from 29 Western All Comers were used; this sample excluded 2 participants of Japanese ancestry that were enrolled in the other drug‐drug interaction cohorts. Data from 18 Western All Comers were used for PK comparisons for 2 × 3000 mg given 12 hours apart.

### Pharmacokinetic Assessments and Analysis

For the single doses, blood samples (3 mL each in the ascending‐dose study or 2 mL each in the to‐be‐marketed formulation study) were collected before dosing and at 0.5, 1, 1.5, 2, 2.5, 3, 4, 6, 8, 12, 24, 36, and 48 hours after dosing. Urine samples were collected before dosing and at intervals of 0‐2, 2‐4, 4‐6, 6‐8, 8‐12, 12‐24, 24‐36, and 36‐48 hours after dosing.

When the study drug was administered as 2 doses 12 hours apart, additional blood and urine samples were collected to assess the PK profile of the second dose. Blood samples (2 mL each) were collected before dosing and at 0.5, 1, 1.5, 2, 2.5, 3, 4, 6, 8, 12, 12.5, 13, 13.5, 14, 14.5, 15, 16, 18, 20, 24, 36, 48, and 60 hours after administration of the first dose. Urine samples were collected before dosing and at intervals of 0‐2, 2‐4, 4‐6, 6‐8, 8‐12, 12‐14, 14‐16, 16‐18, 18‐20, 20‐24, 24‐36, 36‐48, and 48‐60 hours after administration of the first dose.

Plasma and urine concentrations of gepotidacin were determined using validated high‐performance liquid chromatography–tandem mass spectrometry assays at PPD Bioanalytical Laboratory (Middleton, Wisconsin).^14^, ^17^ The plasma and urine assays were validated over the concentration ranges of 0.01‐5.00 and 1.00‐500 µg/mL, respectively. The plasma protein binding of gepotidacin is low (33%); thus, only total plasma drug concentrations were reported.

Standard noncompartmental methods were used to determine gepotidacin plasma and urine PK parameters using Phoenix WinNonlin (Certara USA, Inc., Princeton, New Jersey) version 6.4 (ascending‐dose study) or version 8.0 (to‐be‐marketed formulation study) based on actual sampling times. Plasma PK parameters determined included the area under the plasma concentration–time curve (AUC) from time 0 (before dosing) to time of the last quantifiable concentration (area under the concentration–time curve [AUC]_0–t_), area under the plasma concentration–time curve from time 0 (before dosing) extrapolated to infinite time (AUC_0–∞_), maximum observed concentration (C_max_), time to first occurrence of C_max_ (t_max_), lag time before plasma drug concentrations were observed (t_lag_), and t_1/2_ for both studies; and AUC from time 0 (before dosing) to time τ, where τ = 12 hours (AUC_0–τ_), accumulation ratios (ROs; dose 2/dose 1) for plasma AUC_0–τ_ and C_max_, and the apparent plasma oral clearance were also determined in the to‐be‐marketed formulation study. Dose‐normalized AUC_0–t_ (DNAUC_0–t_), dose‐normalized AUC_0–∞_ (DNAUC_0–∞_), and dose‐normalized C_max_ (DNC_max_) plasma PK parameters and ROs were calculated in the ascending‐dose study. Urine PK parameters included unchanged drug excreted over each collection interval, total unchanged drug excreted in the urine, percentage of the given dose of drug excreted in the urine (fe%), and renal clearance (CLr). Gepotidacin area under the urine concentration–time curve from time 0 (before dosing) to time τ, where τ = 12 hours (AUC_0–τ_), and the area under the urine concentration–time curve from time 0 (before dosing) to 12, 24, and 48 hours after dosing (AUC_0–12_, AUC_0–24_, and AUC_0–48_, respectively) were calculated using the urine concentration–time profile and the linear‐up/log‐down trapezoidal rule.

The minimal number of samples that were not collected, lost, of insufficient volume, or not analyzed were set as missing (no imputation was performed). All PK data are presented as arithmetic mean and standard deviation, unless otherwise indicated. The PK parameters are also presented as geometric mean and coefficient of variation (%CVb; Supporting Information Tables S1–S4). Data are reported as 3 significant digits.

### Statistical Analysis

#### Ascending‐Dose Study

Based on the precision of PK parameters, a sample size of 10 participants was used in part 1 of the study, and a sample size of 12 participants (10 active, 2 placebo) was used in part 2.

The natural log‐transformed PK parameters were evaluated by treatment for outliers using the Grubbs test.^30^ Where applicable, data are presented for all participants and with outliers excluded.

In part 1, dose proportionality under fasted conditions was evaluated using an analysis of variance model with log‐transformed DNAUC_0‐t_, DNAUC_0‐∞_, and DNC_max_ as a dependent variables, dose group as a fixed effect, and participant as a random effect. The model was used to estimate the dose‐normalized geometric means at each dose and the least squares (LS) ratio of geometric means between the 2 doses (3000 versus 1500 mg) with 90%CIs. In part 2, for the 3 gepotidacin doses evaluated under fed conditions, dose proportionality was evaluated using the power model *y* = α × (dose)^β^, where *y* denotes the PK parameter being analyzed (ie, AUC_0‐t_, AUC_0‐∞_, and C_max_).

#### To‐Be‐Marketed Formulation Study

Based on the precision of PK parameters and to allow for up to a 20% withdrawal rate, a sample size of 14 participants (11 active, 3 placebo) was planned to ensure that 12 participants (10 active, 2 placebo) completed the study.

The effect of food on gepotidacin plasma PK exposure was analyzed using a linear mixed‐effect model, with treatment and period as fixed effects and participant as a random effect. Analysis was performed on the natural logarithms of plasma gepotidacin AUC_0‐t_, AUC_0‐∞_, and C_max_ for fed versus fasted conditions. Effects were estimated, and 90%CIs were constructed for the treatment comparison for gepotidacin under fed versus fasted conditions. Point estimates and 90%CIs for treatment differences on the log scale derived from the model were exponentiated to obtain estimates for geometric mean LS ratios and CIs on the original scale. The Kenward‐Roger method for approximating the denominator degrees of freedom and correcting for bias in the estimated variance‐covariance of the fixed effects was used.^31^ A Wilcoxon signed‐rank test was performed to compare plasma t_max_ and t_lag_ across treatments.

Plasma and urine PK comparisons were performed between Japanese and Western (non‐Japanese) participants for a 1500‐mg single dose and for 2 × 3000‐mg doses administered 12 hours apart, both under fed conditions. Interethnic plasma and urine concentration–time profiles and PK parameters were summarized and compared.

### Safety and Tolerability Assessments and Analysis

In both studies, safety and tolerability were assessed through AE collection and review of changes from baseline for clinical laboratory evaluations, vital sign measurements, ECG parameters, and physical examination findings. Holter monitoring was included as an additional safety measure when 2 doses of the study drug were administered 12 hours apart. Descriptive summaries were generated using SAS software version 9.3 or 9.4 (SAS Institute Inc., Cary, North Carolina).

## Results

### Pharmacokinetics

#### Ascending‐Dose Study

**Figure 3:**
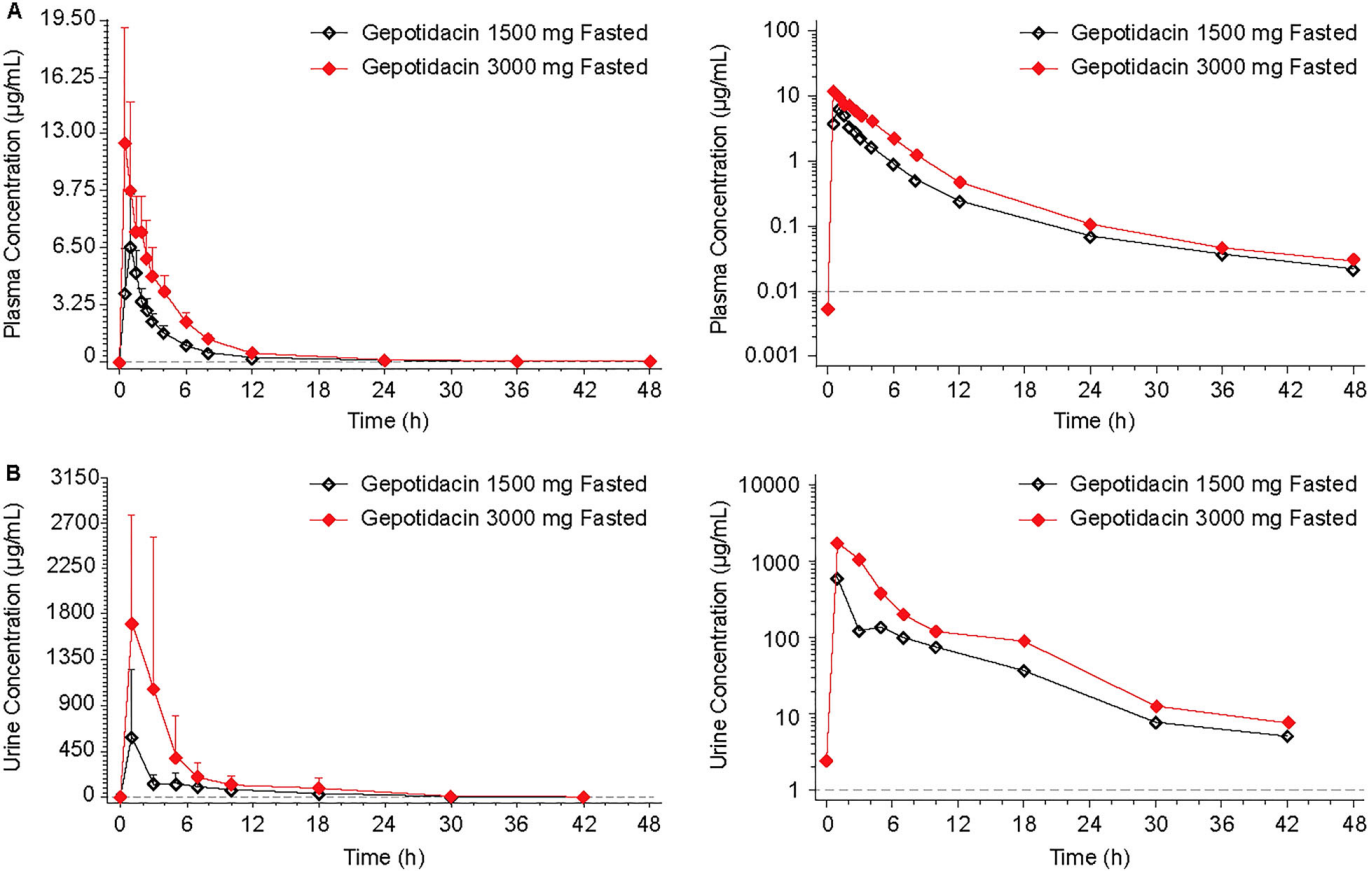
Arithmetic mean (±standard deviation) gepotidacin concentration–time profiles in Japanese participants after administration of 1500‐ and 3000‐mg doses under fasted conditions in plasma with outliers excluded (A) and in urine for all participants (B)—free‐base formulation—ascending dose study. Dashed line represents the lower limit of quantification. For urine, the time points represent the midpoint of the collection interval.

The mean gepotidacin plasma concentration–time profiles for the single 1500‐ and 3000‐mg doses under fasted conditions are shown in Figure 3. After fasted administration of each dose level, gepotidacin was rapidly absorbed, with median t_max_ values of 1 hour (1500 mg) and 0.56 hours (3000 mg) (Table 2). Two outliers were identified using Grubbs analysis; 1 in each dose group (1500 mg: woman, 56.2 kg body weight; 3000 mg: woman, 57.5 kg body weight). Excluding data from those 2 participants decreased the %CVb in C_max_ by 59% for the 1500‐mg dose but no notable difference in C_max_ %CVb was observed for the 3000‐mg dose (Supporting Information Table S1). The %CVb for AUC_0‐∞_ decreased by 11% and 8% for the 1500‐ and 3000‐mg doses, respectively.

**Table 2:** Single‐Dose Plasma Pharmacokinetic Parameters for Gepotidacin in Japanese Participants After Administration Under Fed and Fasted Conditions—Free‐Base Formulation—Ascending‐Dose Study

	Fasted (Part 1)				Fed (Part 2)		
Parameter	1500 mg (N = 10) All Participants	1500 mg (N = 9) Outliers Excluded^a^	3000 mg (N = 10) All Participants	3000 mg (N = 9) Outliers Excluded^a^	1500 mg (N = 10) All Participants	2250 mg (N = 10)	3000 mg (N = 9)
AUC_0‐t_, µg × h/mL	24.0 (9.14)	21.5 (4.45)	47.1 (11.1)	44.1 (5.53)	23.1 (4.16)	37.4 (6.37)	50.8 (10.0)
AUC_0‐∞_, µg × h/mL	24.4 (9.14)	21.8 (4.49)	47.5 (11.1)	44.4 (5.50)	23.3 (4.18)	37.7 (6.38)	51.1 (10.1)
C_max_, µg/mL	14.2 (20.9)	7.67 (2.41)	15.9 (4.35)	15.5 (4.47)	6.73 (1.95)	10.0 (2.24)	13.1 (2.83)
t_max_, h	1.00 (0.50‐2.00)	1.00 (0.50‐1.50)	0.56 (0.50‐2.50)	0.50 (0.50‐2.50)	2.25 (1.00‐4.00)	2.05 (1.50‐4.00)	2.00 (1.00‐3.00)
t_lag_, h	0.00 (0.00‐0.00)	0.00 (0.00‐0.00)	0.00 (0.00‐0.00)	0.00 (0.00‐0.00)	0.50 (0.00‐0.50)	0.00 (0.00‐0.00)	0.00 (0.00‐0.00)
t_1/2_, h	10.8 (1.25)	10.8 (1.32)	8.84 (0.754)	8.97 (0.676)	9.27 (0.924)	8.21 (0.567)	7.97 (0.840)

AUC_0‐∞_, area under the concentration‐time curve from time 0 (before dosing) extrapolated to infinite time; AUC_0‐t_, area under the concentration–time curve from time 0 (before dosing) extrapolated to infinite time; C_max_, maximum observed concentration; SD, standard deviation; t_1/2_, terminal phase half‐life; t_lag_, lag time before plasma drug concentrations were observed; t_max_, time to first occurrence of C_max_.

Values are presented as arithmetic mean (SD), except for t_max_ and t_lag_, which are presented as median (minimum‐maximum).

^a^ Data from 2 participants, 1 from each dose group, were considered outliers based on Grubb analysis and were excluded from the summary. For 1500 mg, the participant excluded had AUC_0‐t_, AUC_0‐∞_, and C_max_ values of 47.1 µg  h/mL, 47.4 µg  h/mL, and 73.3 µg/mL, respectively. For 3000 mg, the participant excluded had AUC_0‐t_, AUC_0‐∞_, and C_max_ values of 74.9 µg  h/mL, 75.4 µg  h/mL, and 18.9 µg/mL, respectively.

A dose‐proportional increase in exposure from 1500 to 3000 mg was observed under fasted conditions for the DNAUCs (DNAUC_0‐t_ and DNAUC_0‐∞_ geometric LS mean ratios: 1.013 and 1.006, respectively, and 90%CIs contained within 0.80–1.25). A similar trend was observed for DNC_max_ although the 90%CIs were outside the specified range for dose proportionality (DNC_max_ geometric mean ratio, 0.835%; and 90%CI: 0.542‐1.287). The DNAUC and DNC_max_ data pattern remained the same when the 2 outliers were excluded.

**Figure 4:**
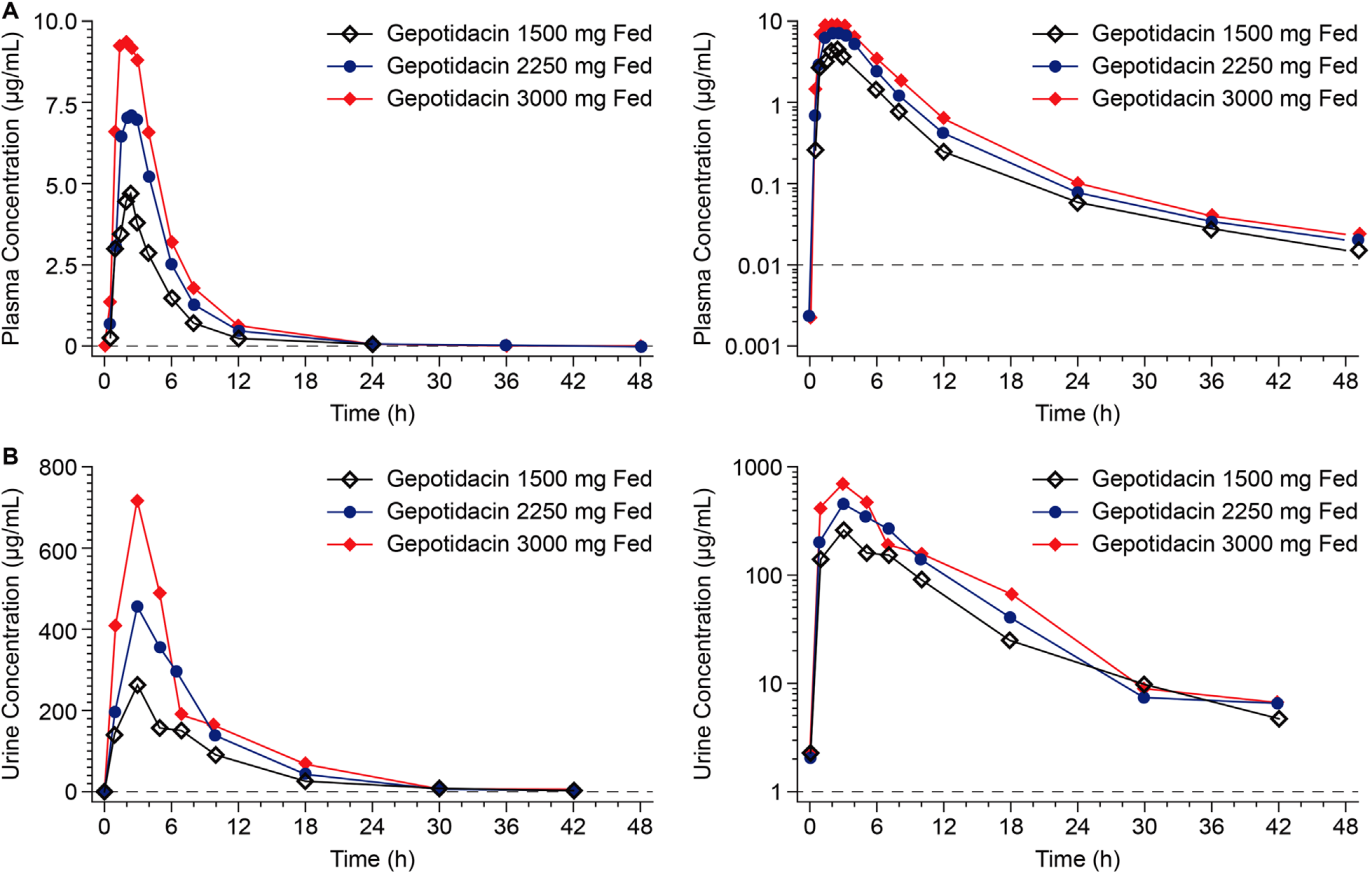
Arithmetic mean gepotidacin concentration–time profiles in Japanese participants after single‐dose administration of 1500, 2250, and 3000 mg under fed conditions in plasma (A) and urine (B)—free‐base formulation—ascending dose study. Dashed line represents the lower limit of quantification. For urine, the time points represent the midpoint of the collection interval.

Under fed conditions, plasma concentrations of gepotidacin increased with dose after single 1500‐, 2250‐, and 3000‐mg doses (Figure 4). The median t_max_ under fed conditions was ≈2 hours across the 3 doses (Table 2). The natural log‐transformed AUC and C_max_ parameter values had an approximate dose proportional increase over the 1500‐3000‐mg dose range, with slopes of 1.152 for AUC_0‐t_, 1.146 for AUC_0‐∞_, and 0.990 for C_max_. The 90%CI lower bound for C_max_ was 0.730; all other 90%CIs were within the bounds of 0.8‐1.25.

For all doses of gepotidacin from 1500 to 3000 mg under either fasted or fed conditions in the ascending‐dose study, mean gepotidacin urine concentrations were measurable through the 36‐ to 48‐hour collection interval (Figures 3 and 4). The CLr was slightly lower following the 1500‐mg (9.61 L/h) compared with the 3000‐mg dose (12.4 L/h) for fasted administration, as also observed for the percent of dose excreted in urine (fe%; 14.5% vs 20.8%) (Table 3). In the fed state, across the 1500–3000‐mg dose range, CLr and fe% were similar for all 3 dose levels.

**Table 3:** Single‐Dose Urine Pharmacokinetic Parameters for Gepotidacin in Japanese Participants After Administration Under Fed and Fasted Conditions—Free‐Base Formulation—Ascending‐Dose Study

	Fasted (Part 1)		Fed (Part 2)		
Parameter	1500 mg (N = 10)	3000 mg (N = 10)	1500 mg (N = 10)	2250 mg (N = 10)	3000 mg (N = 9)
AUC_0‐12_, µg × h/mL	1730 (1180)	5570 (3700)	1720 (775)	2990 (926)	3880 (1640)
AUC_0‐24_, µg × h/mL	2160 (1400)	6460 (4000)	2080 (953)	3540 (1160)	4640 (1940)
AUC_0‐48_, µg × h/mL	2320 (1470)	6730 (4120)	2260 (1070)	3720 (1250)	4850 (2010)
Ae total, mg	217 (79.3)	623 (420)	410 (137)	640 (190)	926 (251)
fe%	14.5 (5.29)	20.8 (14.0)	27.3 (9.13)	28.5 (8.46)	30.9 (8.36)
CLr, L/h	9.61 (3.88)	12.4 (4.50)	17.7 (4.34)	17.1 (4.33)	18.3 (3.86)

Ae total, total unchanged drug excreted in the urine; AUC_0–12_, area under the urine concentration–time curve over time 0 (before dosing) to 12 hours after dosing; AUC_0‐24_, area under the urine concentration–time curve over time 0 (before dosing) to 24 hours after dosing; AUC_0‐48_, area under the urine concentration–time curve over time 0 (before dosing) to 48 hours after dosing; CLr, renal clearance; fe%, percentage of drug excreted; SD, standard deviation.

Values are presented as arithmetic mean (SD).

#### To‐Be‐Marketed Formulation Study

##### Food Effect

**Figure 5:**
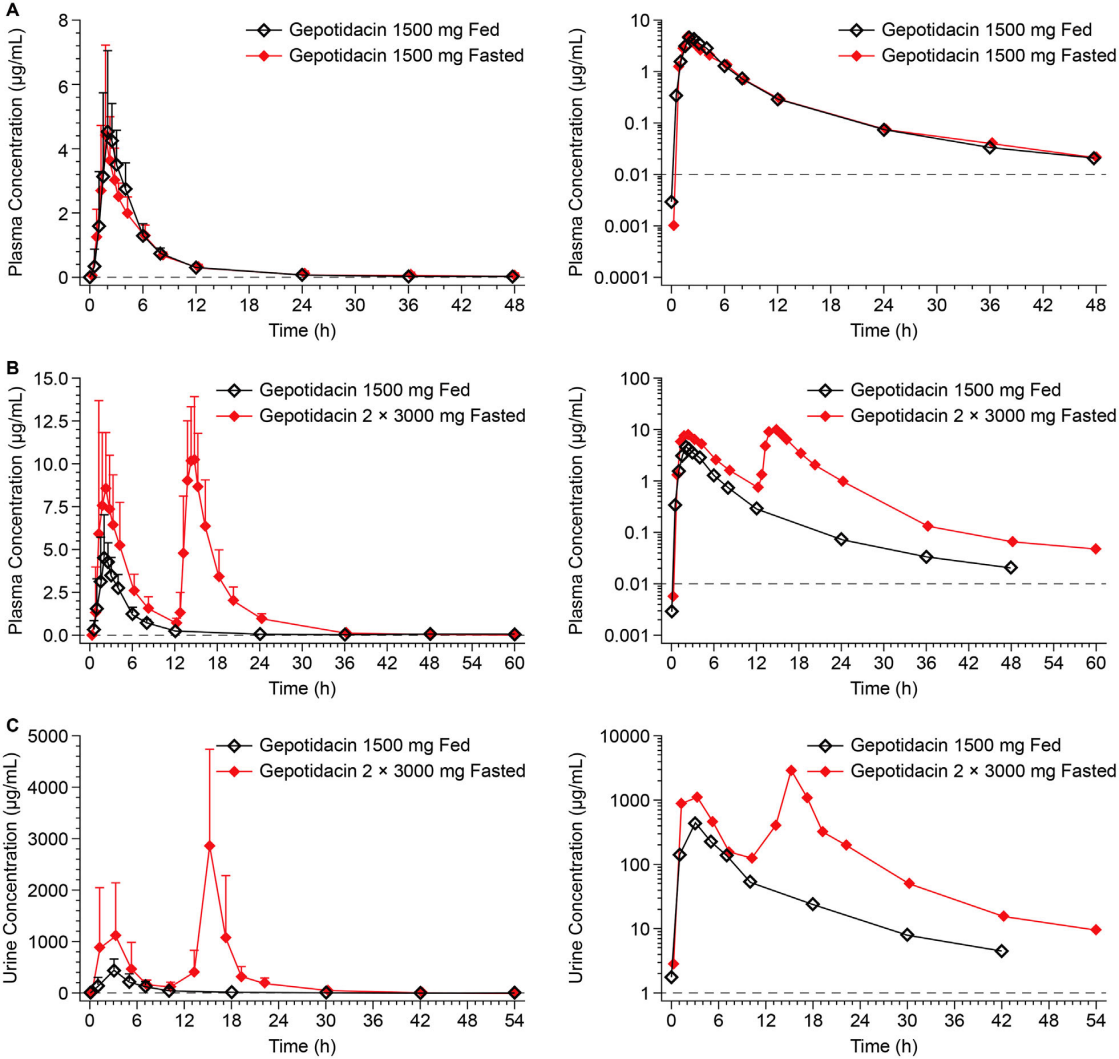
Arithmetic mean (±standard deviation) gepotidacin concentration–time profiles of gepotidacin in Japanese participants after a single 1500‐mg dose administered under fed and fasted conditions in plasma (A), and as a single 1500‐mg dose administered under fed conditions or 2 × 3000‐mg doses given 12 hours apart administered under fed conditions in plasma (B) and urine (C)—mesylate formulation—to‐be‐marketed formulation study. Dashed line represents the lower limit of quantification.

Following a single 1500‐mg gepotidacin dose under fed and fasted conditions, mean gepotidacin plasma concentrations peaked at a median t_max_ of 2.00 and 1.50 hours after dosing, respectively; there was no t_lag_ under either condition (Figure 5 and Table 4). Thereafter, plasma concentrations declined in a similar manner under fed and fasted conditions.

**Table 4:** Plasma Pharmacokinetic Parameters for Gepotidacin Under Fed Conditions – Japanese Versus Western Participants—Mesylate Formulation—To‐Be‐Marketed Formulation Study

	1500‐mg Single Dose^a^			2 × 3000‐mg Doses^b^					
	Western			Western				Japanese (N = 11)	
Parameter	All Comers (N = 29)	White (N = 9)	Japanese (N = 11)	All Comers (N = 18) Dose 1	All Comers (N = 18) Dose 2	White (N = 7) Dose 1	White (N = 7) Dose 2	Dose 1	Dose 2
AUC_0‐t_,^c^µg × h/mL	20.3 (5.96)	21.1 (6.87)	22.1 (3.68)	86.7 (16.7)	…	90.6 (12.7)	…	93.7 (24.9)	…
AUC_0‐∞_, µg × h/mL	20.6 (6.02)	21.5 (6.88)	22.5 (3.60)	…	…	…	…	…	…
AUC_0–τ_, µg × h/mL	…	…	…	31.1 (9.48)	43.4 (10.9)	32.6 (8.51)	47.9 (8.02)	38.5 (11.7)	47.9 (12.5)
AUC_0‐24_,^c^µg × h/mL	19.2 (5.86)	20.1 (6.85)	21.2 (3.71)	75.5 (18.1)	…	81.2 (11.1)	…	86.8 (23.4)	…
AUC_0‐48_,^c^µg × h/mL	20.3 (5.96)	21.1 (6.86)	22.1 (3.68)	83.5 (19.2)	…	89.9 (12.5)	…	93.1 (24.7)	…
C_max_, µg/mL	4.54 (2.10)	5.32 (3.15)	5.63 (1.68)	8.33 (2.85)	11.0 (4.90)	9.33 (3.73)	12.2 (4.86)	12.2 (5.75)	12.6 (2.59)
t_max_, h	2.50 (1.00‐6.00)	2.00 (1.50‐3.02)	2.00 (1.50‐4.00)	2.50 (1.00‐4.07)	2.00 (1.00‐6.00)	2.50 (1.00‐4.00)	2.50 (1.00‐2.77)	2.00 (1.00‐4.00)	2.00 (1.00‐3.00)
t_lag_, h	0.00 (0.00‐1.00)	0.00 (0.00‐0.50)	0.00 (0.00‐0.50)	0.25 (0.00‐1.00)	…	0.50 (0.00‐1.00)	…	0.00 (0.00‐0.00)	…
t_1/2_,^c^, ^d^ h	11.3 (2.53)	10.2 (0.691)	13.3 (3.49)	9.73 (2.28)	…	9.70 (1.99)	…	13.2 (3.88)	…
CL/F,^c^, ^d^ L/h	78.8 (22.9)	76.4 (24.5)	68.0 (10.0)	71.3 (14.7)	…	66.9 (8.66)	…	69.4 (9.22)	…
RO AUC_0‐τ_	…	…	…	…	1.46 (0.406)	…	1.57 (0.537)	…	1.26 (0.166)
RO C_max_	…	…	…	…	1.45 (0.796)	…	1.61 (1.01)	…	1.17 (0.397)

AUC_0‐∞_, area under the concentration‐time curve from time 0 (before dosing) extrapolated to infinite time; AUC_0‐τ_, area under the plasma concentration–time curve from time 0 (before dosing) to time τ, where τ = 12 hours; AUC_0‐24_, area under the plasma concentration–time curve from time 0 (before dosing) to the concentration at 24 hours after dosing; AUC_0‐48_, area under the plasma concentration–time curve from time 0 (before dosing) to the concentration at 48 hours after dosing; AUC_0‐t_, area under the concentration–time curve from time 0 (before dosing) extrapolated to infinite time; CL/F, apparent plasma oral clearance; C_max_, maximum observed concentration; PK, pharmacokinetic; RO, accumulation ratio (dose 2/dose 1) ; SD, standard deviation; t_1/2_, terminal phase half‐life; t_lag_, lag time before plasma drug concentrations were observed; t_max_, time to first occurrence of C_max_.

Values are presented as arithmetic mean (SD), except for t_max_ and t_lag_, which are presented as median (minimum‐maximum). Data for Japanese participants are from the fed 1500 mg dose and 2 × 3000‐mg doses given 12 hours apart. Data for Western participants are from healthy participants who participated in other cohorts of the study; for the 2 × 3000‐mg doses given 12 hours apart, dose 1 was gepotidacin administered alone and dose 2 was gepotidacin coadministered with digoxin and midazolam for a drug‐drug interaction evaluation, with gepotidacin as the potential perpetrator.

^a^ For the 1500 mg dose, comparisons were made with Western All Comers (N = 29) who self‐identified as Black or African American (n = 15), White/Caucasian/European (White; n = 9), Native Hawaiian or Other Pacific Islander (n = 2), or multiple (mixed) ancestries (n = 3). The body weight ranges were as follows: 52‐105.6 kg for n = 29 All Comers, 52‐93.8 kg for n = 9 Whites, and 47.1–80.5 kg for n = 11 Japanese.

^b^ For the 2 × 3000‐mg doses given 12 hours apart, comparisons were made with Western All Comers (N = 18) who self‐identified as Black or African American (n = 11) or White/Caucasian/European (White; n = 7). The body weight ranges were as follows: 57.4–99.9 kg for n = 18 All Comers, 62.4–97.7 kg for n = 7 Whites, and 47.1–80.5 kg for n = 11 Japanese.

^c^ Full profile parameters were calculated using the overall profile for both doses, when applicable.

^d^ n = 17 for 2 × 3000 mg in Western All Comers and n = 10 for 2 × 3000 mg doses in Japanese participants.

Overall the plasma gepotidacin AUC and C_max_ parameters were comparable under fed and fasted conditions in the healthy Japanese participants (Supporting Information Figure S1). The geometric LS mean (90%CI) ratios (gepotidacin 1500 mg fed/gepotidacin 1500 mg fasted) were 1.09 (0.987‐1.21), 1.10 (0.991‐1.21), and 1.05 (0.824‐1.34) for AUC_0‐t_, AUC_0‐∞_, and C_max_, respectively. The 90%CIs for the AUC ratios were within the bounds of 0.80‐1.25, whereas the upper limit of the 90%CI for the C_max_ ratio was above the 1.25 upper boundary limit.

Although urine data was not used to assess a potential food effect for gepotidacin oral absorption, mean gepotidacin urine concentrations were measurable through 48 hours after dosing after a single 1500‐mg dose under fed and fasted conditions. The mean (standard deviation) CLr and fe% were 14.0 L/h (4.00) and 20.3% (5.41), respectively, under fed conditions (Table 5).

**Table 5:** Urine Pharmacokinetic Parameters for Gepotidacin Under Fed Conditions – Japanese Versus Western Participants—Mesylate Formulation—To‐Be‐Marketed Formulation Study

	Single Dose 1500 mg^a^			2 × 3000 mg Doses^b^		
	Western			Western		
Parameter	All Comers (N = 29)	White (N = 9)	Japanese (N = 11)	All Comers (N = 18)	White (N = 7)	Japanese (N = 11)
AUC_0‐24_,^c^ µg × h/mL	3470 (2170)	3190 (2060)	2390 (1110)	16,300 (8070)	20,800 (7780)	16,900 (9150)
AUC_0–48_,^c^ µg × h/mL	3770 (2280)	3410 (2100)	2550 (1170)	19,100 (9610)	24,300 (9500)	18,000 (9590)
AUC_0‐τ_,^c^, ^d^ µg × h/mL	…	…	…	5470 (3320)	6140 (3300)	5830 (3430)
Ae total,^e^ mg	343 (120)	357 (144)	304 (81.1)	1140 (380)	1200 (334)	1370 (348)
fe%^d^, ^e^	22.9 (7.99)	23.8 (9.58)	20.3 (5.41)	19.0 (6.33)	19.9 (5.57)	22.9 (5.80)
CLr,^e^ L/h	16.9 (4.10)	17.0 (3.71)	14.0 (4.00)	13.9 (4.17)	13.3 (3.87)	14.9 (3.02)

Ae total, total unchanged drug excreted in the urine; AUC_0–τ_, area under the urine concentration–time curve from time 0 (before dosing) to time τ, where τ = 12 hours; AUC_0–24_, area under the urine concentration–time curve over time 0 (before dosing) to 24 hours after dosing; AUC_0–48_, area under the urine concentration–time curve over time 0 (before dosing) to 48 hours after dosing; CLr, renal clearance; fe%, percentage of drug excreted; PK, pharmacokinetic; SD, standard deviation.

Values are presented as arithmetic mean (SD). Data for Japanese participants are from the fed 1500 mg dose and 2 × 3000 mg doses given 12 hours apart. Data for Western participants are from healthy participants who participated in other cohorts of the study; for the 2 × 3000 mg doses given 12 hours apart, dose 1 was gepotidacin administered alone and dose 2 was gepotidacin coadministered with digoxin and midazolam for a drug–drug interaction evaluation, with gepotidacin as the potential perpetrator.

^a^ For the 1500 mg dose, comparisons were made with Western All Comers (N = 29) who self‐identified as Black or African American (n = 15), White/Caucasian/European (White; n = 9), Native Hawaiian or Other Pacific Islander (n = 2), or multiple (mixed) ancestries (n = 3). The body weight ranges were as follows: 52–105.6 kg for n = 29 All Comers, 52–93.8 kg for n =9 Whites, and 47.1–80.5 kg for n = 11 Japanese.

^b^ For the 2 × 3000‐mg doses given 12 hours apart, comparisons were made with Western All Comers (N = 18) who self‐identified as Black or African American (n = 11) or White/Caucasian/European (White; n = 7). The body weight ranges were as follows: 57.4–99.9 kg for n = 18 All Comers, 62.4–97.7 kg for n = 7 Whites, and 47.1–80.5 kg for n = 11 Japanese.

^c^ n = 10 for 2 × 3000 mg doses in Japanese participants.

^d^ AUC_0‐τ_ accounted for the first dose and fe% accounted for both doses when 2 doses were administered.

^e^ n = 27 for the 1500‐mg single dose in Western All Comers.

##### Interethnic PK Comparison

**Figure 6:**
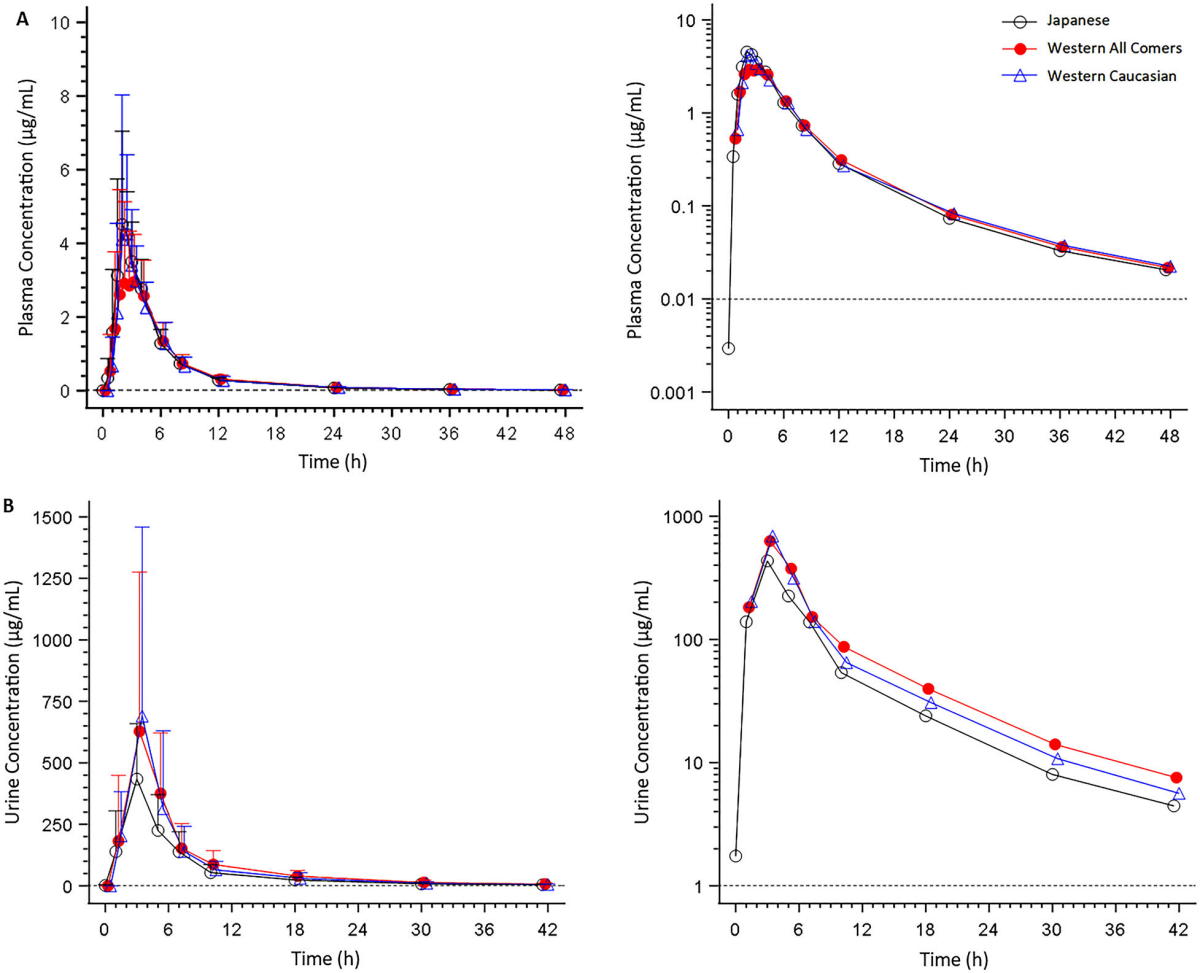
Arithmetic mean (±standard deviation) gepotidacin concentration–time profiles of gepotidacin in Japanese and Western participants after a single 1500‐mg dose administered under fed conditions in plasma (A) and urine (B)—mesylate formulation—to‐be‐marketed formulation study. Dashed line represents the lower limit of quantification. For urine, the time points represent the midpoint of the collection interval. Gepotidacin pharmacokinetic data for Japanese participants is from the fed 1500‐mg single dose based on the data presented herein. Gepotidacin pharmacokinetic data for Western participants are from healthy adult participants who participated in other cohorts of the overall study design and who underwent the same pharmacokinetic procedures as the Japanese participants.

**Figure 7:**
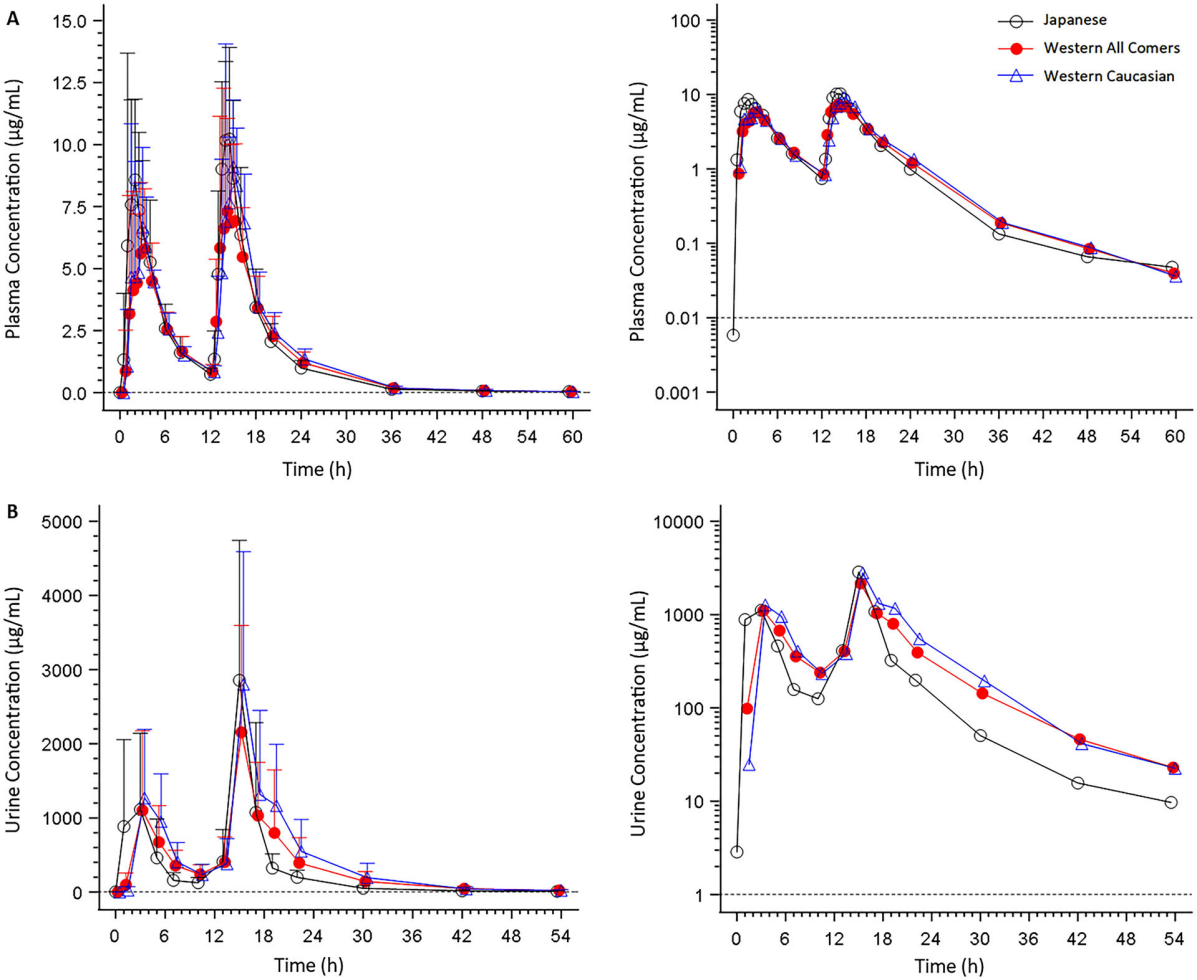
Arithmetic mean (±standard deviation) gepotidacin concentration–time profiles of gepotidacin in Japanese and Western participants administration as 2 × 3000‐mg doses given 12 hours apart under fed conditions in plasma (A) and urine (B)—mesylate formulation—to‐be‐marketed formulation study. Dashed line represents the lower limit of quantification. For urine, the time points represent the midpoint of the collection interval. Gepotidacin PK data for Japanese participants is from the fed 2 × 3000‐mg doses given 12 hours apart based on the data presented herein. Gepotidacin pharmacokinetic data for Western participants are from healthy adult participants who participated in other cohorts of the overall study design and who underwent the same pharmacokinetic procedures as the Japanese participants; for the 2 × 3000‐mg doses given 12 hours apart, dose 1 was gepotidacin administered alone, and dose 2 was gepotidacin coadministered with digoxin and midazolam for a drug–drug interaction evaluation, with gepotidacin as the potential perpetrator. PK, pharmacokinetic.

After single‐dose administration of 1500‐mg gepotidacin under fed conditions, mean plasma concentrations peaked at approximately 2 hours after dosing in healthy Japanese and Western participants, with a similar decline across groups in concentrations following t_max_ (Figure 6). Similar gepotidacin plasma concentrations in healthy Japanese and Western participants were also observed after administration of 2 × 3000‐mg doses given 12 hours apart in the fed state (Figure 7). At both doses, plasma gepotidacin concentrations in the White participants were also similar to those observed in the Japanese participants and the overall cohort of Western participants.

Following a 1500‐mg single dose and 2 × 3000‐mg doses of gepotidacin given 12 hours apart under fed conditions, slightly higher C_max_ values were observed in Japanese participants compared to the subgroup of White participants (15% higher after the 1500‐mg single dose, 30% higher after the first 3000‐mg dose, and 7% higher after the second 3000‐mg dose [geometric mean comparison]) (Table 4 and Supporting Information Table S3). Plasma AUC values were similar in the Japanese, Western, and White participants. Mean gepotidacin t_1/2_ was similar in the Japanese participants and White participants for all treatment conditions (dose, fed/fasted). After administration of 2 × 3000‐mg doses of gepotidacin, limited accumulation was observed in the Japanese and White participants (lower RO AUC_0‐τ_ 1.26 vs 1.57 and lower RO C_max_ 1.17 vs 1.61, respectively).

Following administration of a single 1500‐mg or 2 × 3000‐mg gepotidacin doses, mean urine concentrations were highest during the 2‐ to 4‐hour postdose collection interval in the Japanese, Western, and White participants. In all 3 groups, there was a similar decline in urine concentrations thereafter (Figure 7).

Gepotidacin urine PK parameters were comparable in the Japanese, Western, and White participants for all treatment conditions, with slightly higher urine AUCs in the White participants than in the Japanese or Western participants for the 2 × 3000‐mg gepotidacin doses (Table 5).

### Safety and Tolerability

#### Ascending‐Dose Study

At the 1500‐ and 3000‐mg single doses, 9 (90%) and 6 (50%) Japanese participants experienced at least 1 AE under fasted and fed conditions, respectively (Table 6). At the 1500‐mg dose, no participants reported AEs under fed conditions compared with 60% of participants in the fasted state. AEs were reported only in the system organ classes of gastrointestinal (GI) disorders (90% incidence fasted; 50% incidence fed) and nervous system disorders (70% incidence fasted; 17% incidence fed) (Table 6). The AEs with the highest prevalence were diarrhea and nausea for the fasted state, and nausea, abdominal discomfort, and soft feces for the fed state.

**Table 6:** Summary of Adverse Events Reported in at Least 2 Japanese Participants in Each Study Part or Overall After Gepotidacin or Placebo Administration Under Fed and Fasted Conditions

	Ascending‐Dose Study^a^ Part 1, Fasted			Ascending‐Dose Study^a^ Part 2, Fed						To‐Be‐Marketed Formulation Study^b^			
System Organ Class Preferred Term, n (%)	1500 mg (N = 10)	3000 mg (N = 10)	Total (N = 10)	1500 mg (N = 10)	2250 mg (N = 10)	3000 mg (N = 9)	Placebo (N = 2)	Total (N = 12)	Placebo (N = 3)	Fasted 1500 mg (N = 11)	Fed 1500 mg (N = 11)	Fed 3000 mg^c^ (N = 11)	Total (N = 14)
Any event	6 (60)	9 (90)	9 (90)	0	1 (10)	6 (67)	0	6 (50)	0	3 (27)	1 (9)	4 (36)	5 (36)
Gastrointestinal disorders	5 (50)	9 (90)	9 (90)	0	1 (10)	6 (67)	0	6 (50)	0	2 (18)	1 (9)	4 (36)	5 (36)
Abdominal discomfort	0	0	0	0	0	2 (22)	0	2 (17)	0	0	0	0	0
Diarrhea	5 (50)	9 (90)	9 (90)	0	0	1 (11)	0	1 (8)	0	0	0	3 (27)	3 (21)
Feces soft	0	0	0	0	1 (10)	2 (22)	0	2 (17)	0	0	0	0	0
Nausea	0	6 (60)	6 (60)	0	0	4 (44)	0	4 (33)	0	2 (18)	1 (9)	2 (18)	3 (21)
Vomiting	0	5 (50)	5 (50)	0	0	1 (11)	0	1 (8)	0	1 (9)	0	1 (9)	2 (14)
Nervous system disorders	3 (30)	7 (70)	7 (70)	0	0	2 (22)	0	2 (17)	0	0	0	1 (9)	1 (7)
Dizziness	1 (10)	4 (40)	4 (40)	0	0	1 (11)	0	1 (8)	0	0	0	1 (9)	1 (7)
Headache	2 (20)	3 (30)	4 (40)	0	0	2 (22)	0	2 (16)	0	0	0	0	0

The data represent the number of participants with the adverse event, not the number of events. At each level of summarization, a participant was counted once.

^a^ Free‐base formulation.

^b^ Mesylate formulation.

^c^ Gepotidacin was administered as 2 × 3000‐mg doses given 12 hours apart.

AEs considered moderate in severity by the investigator included 2 events of drug‐related diarrhea, 1 event of drug‐related vomiting, and 1 event of non–drug‐related headache (all 3000‐mg dose, fasted condition). All other AEs were mild. No changes from baseline or findings for vital signs, laboratory evaluations, or ECG parameters were of clinical concern.

#### To‐Be‐Marketed Formulation Study

Overall, 5 Japanese participants (36%) experienced at least 1 AE. When 1500‐mg gepotidacin was administered in the fed and fasted states, 1 participant (9%) and 3 participants (27%) reported AEs, respectively (Table 6). AEs were most prevalent in the GI disorders system organ class, most commonly diarrhea and nausea. All AEs reported were considered mild, including an AE of first‐degree atrioventricular block that was considered unrelated to the study drug by the investigator. No changes from baseline or findings in the other safety parameters were of clinical concern. There were no new safety findings compared with earlier phase 1 studies.^13^, ^14^, ^15^, ^16^, ^17^, ^18^

## Discussion

The phase 1 studies presented herein were conducted to characterize oral gepotidacin PK, safety, and tolerability in healthy Japanese participants. The free‐base formulation of gepotidacin initially studied showed an approximate dose‐proportional increase in healthy Japanese participants from 1500 to 3000 mg for plasma AUC and C_max_ when fed and fasted. The free‐base formulation in the initial study was later modified to a mesylate formulation; previous evaluations demonstrated that the free‐base and mesylate formulations provide comparable systemic exposures.^18^ Due to this change in formulation, a second PK study was conducted in healthy Japanese participants to assess the PK profile of the to‐be‐marketed mesylate formulation used in the phase 3 program. In the Japanese participants, the gepotidacin mesylate tablet formulation was shown to have similar exposures under fed and fasted conditions after a single 1500‐mg dose. The 90%CIs for plasma AUCs were contained within the 0.80‐1.25 bounds, while the upper limit of the 90%CI for C_max_ (0.824‐1.34) was >1.25. Overall, there was no clinically relevant effect of food on the plasma systemic exposure of oral gepotidacin.

The oral gepotidacin doses characterized in healthy Japanese participants, 1500 mg and 2 × 3000 mg given 12 hours apart, align with those used in the current phase 3 studies. The phase 3 uUTI dose regimen was based on achieving the target free drug AUC_0‐24_/minimum inhibitory concentration (*f*AUC/MIC) of 13, which is the PK/pharmacodynamic index and magnitude that best predicts gepotidacin efficacy in uUTI.^12^, ^32^, ^33^, ^34^ In the phase 3 uUTI studies, 1500‐mg gepotidacin is administered twice daily for 5 days.

In the studies in healthy Japanese participants, after single‐dose administration under fed and fasted conditions, mean gepotidacin urine concentrations ≥4 µg/mL were achieved and maintained for ≥24 hours after dosing in both cohorts. Mean urine gepotidacin concentrations of ≈50 µg/mL at around 12 hours after dosing (twice‐daily dosing for uUTI) were observed in Japanese participants (Figure 6). Gepotidacin has an MIC at which 90% of a sample of isolates are inhibited of 4 µg/mL against quinolone‐resistant *E coli*,^1^ a predominant uropathogen in uUTI, which further indicates the clinical importance of gepotidacin urine concentrations ≥4 µg/mL. In addition to the *f*AUC/MIC, trough gepotidacin urine concentrations are an important threshold value for gepotidacin microbiological efficacy, as the urine/bladder is the site of action for a uUTI indication.^12^

In uncomplicated urogenital gonorrhea, based on a phase 2 clinical study^9^, ^10^ and PK/pharmacodynamic modeling, the phase 3 dose regimen is 2 × 3000‐mg doses given 10‐12 hours apart. This regimen was selected to provide gepotidacin systemic exposures that would maximize efficacy and prevent amplification of resistance against *N gonorrhoeae* isolates with gepotidacin MICs up to 1 µg/mL while also managing systemic exposures that may affect safety and tolerability.^11^ Our study demonstrated similar gepotidacin plasma AUCs in Japanese and Western participants for this 2‐dose regimen, which suggests a comparable *f*AUC/MIC against *N gonorrhoeae* would be attained across populations.

Comparisons were performed between Japanese and Western (non‐Japanese) participants to assess relative gepotidacin exposures in plasma and urine. The to‐be‐marketed formulation study design included 3 additional cohorts of healthy participants of Black African, White, Native Hawaiian, or multiple (mixed) ancestries (NCT04493931/manuscript in preparation). Therefore, the design allowed for within‐study comparisons of gepotidacin PK in Japanese and non‐Japanese participants, or just the White participants investigated under the same study conditions. Overall, slightly higher plasma C_max_ values (7%‐30%) and slightly lower urine AUC values (11%‐18%) were observed across both doses in Japanese relative to White participants. The 30% higher C_max_ observed after the first 3000‐mg dose is partly due to a notably higher C_max_ value observed in 1 Japanese participant (27.0 µg/mL, 34‐year‐old woman, body weight 54.8 kg), which was substantially higher than the median and arithmetic mean data from the other Japanese participants (11.5 and 12.2 µg/mL, respectively). The trend to slightly higher plasma C_max_ values in the healthy Japanese than in White participants can reflect interethnic differences in the factors known to influence gepotidacin PK. As noted previously, gepotidacin is primarily metabolized by CYP3A4, and the major route of elimination following oral and intravenous administration is as unchanged gepotidacin in urine.^13^ The median activity of CYP3A4 is similar in healthy populations of Japanese and White ancestry,^23^ indicating that similar values of PK parameters related to CYP3A4 metabolism are anticipated. Initial population PK analyses indicate that body weight is a significant predictor of gepotidacin apparent clearance, and consequently the trend to slightly higher C_max_ values in Japanese than White participants could reflect the interethnic differences in body weight observed. The mechanisms of active transport for gepotidacin renal clearance have not been fully elucidated, as in vitro studies only identified MATE, yet a clinical drug‐drug interaction study with cimetidine (nonspecific organic cation transporter 2/MATE inhibitor) did not affect gepotidacin PK, suggesting that other transport proteins are involved in the active secretion process (NCT04493931/manuscript in preparation). Therefore, the mechanism responsible for the limited interethnic difference in gepotidacin urine AUC observed is not clear. As noted previously, urine concentrations above the MIC considered to be of clinical relevance were observed in the Japanese and non‐Japanese participants, indicating that a limited difference in gepotidacin urinary excretion is not expected to be of clinical relevance for efficacy.

It is ideal to perform interethnic PK comparisons using data obtained in a single study as differences in study conduct can contribute to any difference and variability observed. Nevertheless, the PK parameters of gepotidacin observed in plasma and urine in the healthy Japanese in both studies and the Western participants in the second study are generally similar to those observed in other clinical studies reporting gepotidacin PK at 1500 and 3000 mg.^16^, ^17^, ^18^

The overall safety profile of oral gepotidacin in healthy Japanese participants was similar to previous phase 1 studies that evaluated oral dosing in the fed or fasted state or intravenous dosing.^13^, ^14^, ^15^, ^16^, ^17^, ^18^ As expected, the greatest incidence of AEs was in the GI system organ class. In the ascending‐dose study, a single 1500‐mg dose in the fasted state had generally good GI tolerability (ie, 50% of participants reported at least 1 GI AE), and AEs were predominantly mild, while there was a distinguishable decrease in GI tolerability with the 3000‐mg dose (ie, 90% of participants reported at least 1 GI AE). However, at 1500, 2250, and 3000‐mg doses under fed conditions, GI tolerability was much improved; 0 (0%), 1 (10%), and 6 (67%) participants reported GI AEs at the 1500, 2250, and 3000 mg doses, respectively. All AEs reported in the Japanese participants under fed conditions were considered mild. Therefore, the GI tolerability was associated with increased dose and can be reduced with the presence of food. Despite the small number of Japanese participants, these results have added to the weight of evidence to inform dosing instructions in subsequent clinical studies, with each gepotidacin dose to be taken with food. It is important to recognize that gepotidacin inhibits acetylcholinesterase, which confounds interpretation of the GI AE profile observed and limits options other than administration with food and dose to manage GI tolerability.

A phase 1 study on cardiac conduction showed a peak effect on Fridericia's corrected QT interval (QTcF; 20‐millisecond prolongation) at average gepotidacin concentrations of 13.3 µg/mL (90%CI, 12.7‐13.9) after intravenous infusion of 1800 mg of gepotidacin^15^; thus, dose levels are cautiously chosen for each clinical study to minimize C_max_ values ≥14 µg/mL. In the healthy Japanese participants in the to‐be‐marketed gepotidacin tablet formulation study, mean C_max_ values of the 1500‐mg single dose and 2 × 3000‐mg doses were below this threshold. Two participants had C_max_ values ≥14 µg/mL after the first dose, and 2 participants after the second dose, with the 2 × 3000‐mg dose, but without associated ECG parameter findings (ie, no QTcF >500 milliseconds and no QTcF changes from baseline ≥60 milliseconds). No C_max_ excursions ≥14 µg/mL were observed for the 1500‐mg dose under phase 3 evaluation for uUTI.

## Conclusion

These 2 clinical studies characterized the gepotidacin PK, safety, and tolerability profile in healthy Japanese participants, which bridged the previous knowledge gap for this ethnic group. The to‐be‐marketed gepotidacin mesylate formulation was studied in Japanese participants as a single 1500‐mg dose under fed and fasted conditions and as 2 × 3000‐mg doses given 12 hours apart under fed conditions, with demonstrated urine and plasma exposures that are suggestive of efficacy in uUTI and uncomplicated urogenital gonorrhea. Linear PK was observed across the 1500‐ to 3000‐mg dose range in fed and fasted states in Japanese participants. Comparable plasma AUC but slightly higher C_max_ (7%‐30%) was observed in Japanese participants compared with non‐Japanese participants, which is considered to be related to differences in body weight. The AE profile of gepotidacin in Japanese participants was comparable with previous phase 1 evaluations, with a predominance of GI AEs reported. When gepotidacin was administered with food, there was notable improvement in GI tolerability. Overall, the gepotidacin PK and safety‐risk profiles in healthy Japanese participants support potential evaluation of the global clinical doses in future studies.

## Conflicts of Interest

Darin B. Brimhall was the principal investigator for the to‐be‐marketed formulation study. Caroline R. Perry, Annette S. Gross, Shaila Shabbir, Sebin Thomas, Meenakshi Srinivasan, and Brandon Swift are employees of and own stock in GSK. Aline Barth, Mohammad Hossain, and Etienne F. Dumont are former employees of GSK and hold company stock. Hirofumi Ogura is a former employee of GSK. Authors were not paid for their manuscript contributions.

## Funding

This study was supported by GSK (Collegeville, PA). This work was also supported in whole or in part with federal funds from the Office of the Assistant Secretary for Preparedness and Response, Biomedical Advanced Research and Development Authority (HHSO100201300011C). Medical writing assistance was provided by Jodi Stahlman (PPD, part of Thermo Fisher Scientific) and Joanna Wilson (Ashfield MedComms, an Inizio company) funded by GSK.

## Supporting information



Supporting Information
