# Supplementary material for: Pharmacokinetic, Safety, and Tolerability Evaluations of Gepotidacin (GSK2140944) in Healthy Japanese Participants
Source: Clin Pharmacol Drug Dev. 2022 Dec 5;12(1):38–56. doi: 10.1002/cpdd.1192 (PMC10107257; doi:10.1002/cpdd.1192)
Supplement: Supplementary file 1 — Supporting Information [file CPDD-12-38-s001.docx]

**Supplemental Materials**

**Pharmacokinetic, Safety, and Tolerability Evaluations of Gepotidacin (GSK2140944) in Healthy Japanese Participants**

Barth et al 2022

**Table S1** Single Dose Plasma Pharmacokinetic Parameters for Gepotidacin in Japanese Participants After Administration Under Fed and Fasted Conditions – Free Base Formulation – Ascending Dose Study

**Table S2** Single Dose Urine Pharmacokinetic Parameters for Gepotidacin in Japanese Participants After Administration Under Fed and Fasted Conditions – Free Base Formulation – Ascending Dose Study

**Table S3** Plasma Pharmacokinetic Parameters for Gepotidacin Under Fed Conditions – Japanese Versus Western Participants – Mesylate Formulation – To‑Be‑Marketed Formulation Study

**Table S4** Urine Pharmacokinetic Parameters for Gepotidacin Under Fed Conditions – Japanese Versus Western Participants – Mesylate Formulation – To‑Be‑Marketed Formulation Study

**Figure S1** Geometric mean and individual plasma AUC_0-t_ (A), AUC_0-∞_ (B), and C_max_ (C) of gepotidacin in Japanese participants after administration of a single 1500 mg dose (mesylate formulation) under fed and fasted conditions – mesylate formulation – to-be-marketed formulation study.

Bottom and top box lines represent the lower and upper quartiles, Q1 and Q3, respectively, with the median represented by the middle box line. Top and bottom whiskers present the maximum and minimum values, respectively. Geometric mean is denoted by a closed green circle.

**Table S1.** Single Dose Plasma Pharmacokinetic Parameters for Gepotidacin in Japanese Participants After Administration Under Fed and Fasted Conditions – Free Base Formulation – Ascending Dose Study

| Parameter |  | Fasted (Part 1) | | | | |  | | Fed (Part 2) | | | | |  |
| --- | --- | --- | --- | --- | --- | --- | --- | --- | --- | --- | --- | --- | --- | --- |
|  |  | 1500 mg  (N = 10) | | 1500 mg  (N = 9) | 3000 mg  (N = 10) | 3000 mg  (N = 9) |  | | 1500 mg  (N = 10) | 2250 mg  (N = 10) | 3000 mg  (N = 9) | | |  |
|  |  | All Participants | | Outliers Excluded^a^ | All Participants | Outliers Excluded^a^ |  | | All Participants | | | | |  |
| AUC_0-t_ (µg•h/mL) |  | 22.8 (34.4) | | 21.0 (23.3) | 46.1 (21.1) | 43.7 (13.0) |  | | 22.7 (20.0) | 36.9 (17.2) | 49.8 (21.7) | | |  |
| AUC_0-∞_ (µg•h/mL) |  | 23.1 (34.1) | | 21.4 (23.0) | 46.5 (20.9) | 44.1 (12.6) |  | | 22.9 (19.9) | 37.2 (17.2) | 50.2 (21.9) | | |  |
| C_max_ (µg/mL) |  | 9.18 (94.8) | | 7.29 (36.1) | 15.3 (28.6) | 15.0 (29.3) |  | | 6.50 (27.7) | 9.81 (21.1) | 12.9 (21.1) | | |  |
| t_max_ (h) |  | 1.00 (0.50-2.00) | | 1.00 (0.50-1.50) | 0.56 (0.50-2.50) | 0.50 (0.50-2.50) |  | | 2.25 (1.00-4.00) | 2.05 (1.50-4.00) | 2.00 (1.00-3.00) | | |  |
| t_lag_ (h) |  | 0.00 (0.00-0.00) | | 0.00 (0.00-0.00) | 0.00 (0.00-0.00) | 0.00 (0.00-0.00) |  | | 0.50 (0.00-0.50) | 0.00 (0.00-0.00) | 0.00 (0.00-0.00) | | |  |
| t_1/2_ (h) |  | 10.7 (10.9) | | 10.7 (11.5) | 8.81 (8.4) | 8.94 (7.4) |  | | 9.23 (9.7) | 8.19 (7.2) | 7.93 (10.6) | | |  |
| %CVb, inter-participant variability; AUC_0-∞_, area under the concentration-time curve from time 0 (predose) extrapolated to infinite time; AUC_0-t_, area under the concentration-time curve from time 0 (predose) extrapolated to infinite time; C_max_, maximum observed concentration; t_lag_, lag time before plasma drug concentrations were observed; t_max_, time to first occurrence of C_max_; t_1/2_, terminal phase half-life; t_max_, time to first occurrence of C_max_.  Values are presented as geometric mean (%CVb), except for t_max_ and t_lag_, which are presented as median (minimum-maximum).  ^a^ Data from 2 participants, one from each dose group, were considered outliers based on Grubb’s analysis and were excluded from the summary. For 1500 mg, the participant excluded had AUC_0-t_, AUC_0-∞_, and C_max_ values of 47.1 µg•h/mL, 47.4 µg•h/mL, and 73.3 µg/mL, respectively. For 3000 mg, the participant excluded had AUC_0-t_, AUC_0-∞_, and C_max_ values of 74.9 µg•h/mL, 75.4 µg•h/mL, and 18.9 µg/mL, respectively. | | | | | | | | | | | | | |  |
|  | | |  | | | |  |  | | | |  |  | |

**Table S2.** Single Dose Urine Pharmacokinetic Parameters for Gepotidacin in Japanese Participants After Administration Under Fed and Fasted Conditions – Free Base Formulation – Ascending Dose Study

| Parameter |  | Fasted (Part 1) | |  | Fed (Part 2) | | |
| --- | --- | --- | --- | --- | --- | --- | --- |
|  |  | 1500 mg (N = 10) | 3000 mg (N = 10) |  | 1500 mg (N = 10) | 2250 mg (N = 10) | 3000 mg (N = 9) |
| AUC_0-12_ (µg•h/mL) |  | 1300 (111) | 4760 (64.6) |  | 1570 (48.3) | 2810 (42.4) | 3520 (52.4) |
| AUC_0-24_ (µg•h/mL) |  | 1710 (88.8) | 5590 (61.9) |  | 1880 (51.8) | 3300 (45.5) | 4200 (54.1) |
| AUC_0-48_ (µg•h/mL) |  | 1880 (83.6) | 5850 (61.1) |  | 2020 (54.3) | 3460 (46.6) | 4390 (54.1) |
| Ae total (mg) |  | 204 (38.8) | 546 (51.8) |  | 390 (34.3) | 614 (32.2) | 896 (27.8) |
| fe% (%) |  | 13.6 (38.8) | 18.2 (51.8) |  | 26.0 (34.3) | 27.3 (32.2) | 29.9 (27.8) |
| CLr (L/hr) |  | 8.95 (41.5) | 11.8 (32.0) |  | 17.2 (24.8) | 16.6 (26.5) | 18.0 (21.2) |
| %CVb, inter-participant variability; _-12_, area under the urine concentration-time curve over time 0 (predose) to 12 hours after dosing; AUC_0-24_, area under the urine concentration-time curve over time 0 (predose) to 24 hours after dosing; AUC_0-48_, area under the urine concentration‑time curve over time 0 (predose) to 48 hours after dosing; Ae total, total unchanged drug excreted in the urine; CLr, renal clearance; fe%, percentage of drug excreted.  Values are presented as geometric mean (%CVb). | | | | | | | |

**Table S3.** Plasma Pharmacokinetic Parameters for Gepotidacin Under Fed Conditions – Japanese Versus Western Participants – Mesylate Formulation – To‑Be‑Marketed Formulation Study

| Parameter | |  | 1500 mg Single Dose | |  | 1500 mg Single Dose |  | 2 × 3000 mg Doses | | | |  | 2 × 3000 mg Doses | |
| --- | --- | --- | --- | --- | --- | --- | --- | --- | --- | --- | --- | --- | --- | --- |
|  |  |  | Western | |  | Japanese (N = 11) |  | Western | | | |  | Japanese (N = 11) | |
|  |  |  | All Comers  (N = 29) | Caucasian (N = 9) |  |  |  | All Comers (N = 18) | All Comers (N = 18) | Caucasian (N = 7) | Caucasian (N = 7) |  |  |  |
|  |  | | | | | |  | Dose 1 | Dose 2 | Dose 1 | Dose 2 |  | Dose 1 | Dose 2 |
| AUC_0-t_^a^ (µg•h/mL) | |  | 19.4 (29.8) | 20.2 (32.9) |  | 21.9 (16.0) |  | 85.2 (20.1) | – | 89.8 (13.5) | – |  | 91.4 (23.0) | – |
| AUC_0-∞_ (µg•h/mL) | |  | 19.8 (29.6) | 20.5 (32.6) |  | 22.3 (15.5) |  | – | – | – | – |  | – | – |
| AUC_0-τ_ (µg•h/mL) | |  | – | – |  | – |  | 29.8 (30.8) | 41.9 (30.2) | 31.6 (27.5) | 47.2 (18.3) |  | 37.3 (25.4) | 46.7 (23.1) |
| AUC_0-24_^a^ (µg•h/mL) | |  | 18.4 (30.8) | 19.1 (34.4) |  | 20.9 (16.8) |  | 73.2 (26.8) | – | 80.6 (13.3) | – |  | 84.6 (23.1) | – |
| AUC_0-48_^a^ (µg•h/mL) | |  | 19.5 (29.8) | 20.2 (32.9) |  | 21.9 (16.0) |  | 81.2 (25.9) | – | 89.2 (13.4) | – |  | 90.8 (22.9) | – |
| C_max_ (µg/mL) | |  | 4.20 (40.9) | 4.71 (53.6) |  | 5.44 (27.8) |  | 7.87 (36.8) | 10.1 (47.7) | 8.60 (48.0) | 11.6 (33.3) |  | 11.2 (45.0) | 12.4 (21.3) |
| t_max_ (h) | |  | 2.50 (1.00-6.00) | 2.00 (1.50-3.02) |  | 2.00 (1.50-4.00) |  | 2.50 (1.00-4.07) | 2.00 (1.00-6.00) | 2.50 (1.00-4.00) | 2.50 (1.00-2.77) |  | 2.00 (1.00-4.00) | 2.00 (1.00-3.00) |
| t_lag_ (h) | |  | 0.00 (0.00-1.00) | 0.00 (0.00-0.50) |  | 0.00 (0.00-0.50) |  | 0.25 (0.00-1.00) | – | 0.50 (0.00-1.00) | – |  | 0.00 (0.00-0.00) | – |
| t_1/2_^a,b^ (h) | |  | 11.1 (22.4) | 10.2 (6.8) |  | 12.8 (28.5) |  | 9.50 (22.5) | – | 9.53 (20.7) | – |  | 12.6 (36.6) | – |
| CL/F^a,b^ (L/h) | |  | 75.7 (29.6) | 73.1 (32.6) |  | 67.3 (15.5) |  | 70.0 (20.1) | – | 66.4 (13.5) | – |  | 68.8 (13.9) | – |
| RO C_max_ | |  | – | – |  | – |  | – | 1.28 (54.4) | – | 1.35 (70.2) |  | – | 1.10 (39.3) |
| RO AUC_0-τ_ | |  | – | – |  | – |  | – | 1.41 (27.5) | – | 1.50 (35.9) |  | – | 1.25 (13.1) |
| %CVb, inter-participant variability; AUC_0-∞_, area under the concentration-time curve from time 0 (predose) extrapolated to infinite time; AUC_0-τ_, area under the plasma concentration-time curve from time 0 (predose) to time τ, where τ = 12 hours; AUC_0-24_, area under the plasma concentration-time curve from time 0 (predose) to the concentration at 24 hours postdose; AUC_0-48_, area under the plasma concentration-time curve from time 0 (predose) to the concentration at 48 hours postdose; AUC_0-t_, area under the concentration-time curve from time 0 (predose) extrapolated to infinite time; CL/F, apparent plasma oral clearance; C_max_, maximum observed concentration; PK, pharmacokinetic; RO, accumulation ratio (dose 2/dose 1); t_1/2_, terminal phase half-life; t_lag_, lag time before plasma drug concentrations were observed; t_max_, time to first occurrence of C_max_.  Values are presented as geometric mean (%CVb), except for t_max_ and t_lag_, which are presented as median (minimum-maximum). Gepotidacin PK data for Japanese participants is from the fed 1500 mg single dose and fed 2 × 3000 mg doses given 12 hours apart from the data presented herein. Gepotidacin PK data for Western participants are from healthy adult participants who participated in other cohorts of the overall study design and who underwent the same PK procedures as the Japanese participants; for the 2 × 3000 mg doses given 12 hours apart, dose 1 was gepotidacin administered alone and dose 2 was gepotidacin co-administered with digoxin and midazolam for a drug-drug interaction evaluation, with gepotidacin as the potential perpetrator. Refer to the respective main manuscript table for Western participant demographics.  ^a^ Full profile parameters were calculated using the overall profile for both doses, when applicable.  ^b^ n = 17 for 2 × 3000 mg in Western All Comers and n = 10 for 2 × 3000 mg doses in Japanese participants. | | | | | | | | | | | | | | |

**Table S4.** Urine Pharmacokinetic Parameters for Gepotidacin Under Fed Conditions – Japanese Versus Western Participants – Mesylate Formulation – To‑Be‑Marketed Formulation Study

| Parameter |  | Single Dose 1500 mg | |  | Single Dose 1500 mg |  | 2 × 3000 mg Doses | |  | 2 × 3000 mg Doses |
| --- | --- | --- | --- | --- | --- | --- | --- | --- | --- | --- |
|  |  | Western | |  | Japanese (N = 11) |  | Western | |  | Japanese (N = 11) |
|  |  | All Comers (N = 29) | Caucasian (N = 9) |  |  |  | All Comers (N = 18) | Caucasian (N = 7) |  |  |
| AUC_0-24_^a^ (µg•h/mL) |  | 2970 (60.1) | 2620 (74.9) |  | 2140 (53.5) |  | 14300 (59.2) | 19200 (48.7) |  | 14700 (59.3) |
| AUC_0-48_^a^ (µg•h/mL) |  | 3250 (58.2) | 2850 (70.9) |  | 2290 (53.2) |  | 16700 (59.4) | 22300 (51.6) |  | 15800 (58.8) |
| AUC_0-τ_^a,b^ (µg•h/mL) |  | – | – |  | – |  | 4770 (55.2) | 5620 (43.7) |  | 5000 (64.4) |
| Ae total^c^ (mg) |  | 324 (36.3) | 337 (35.5) |  | 294 (29.0) |  | 1070 (40.4) | 1150 (32.7) |  | 1330 (25.4) |
| fe%^b,c^ (%) |  | 21.6 (36.3) | 22.4 (35.5) |  | 19.6 (29.0) |  | 17.8 (40.4) | 19.1 (32.7) |  | 22.2 (25.4) |
| CLr^c^ (L/h) |  | 16.4 (26.6) | 16.7 (22.6) |  | 13.4 (31.9) |  | 13.2 (34.6) | 12.8 (30.0) |  | 14.6 (19.3) |
| %CVb, inter-participant variability; AUC_0-τ_, area under the urine concentration-time curve from time 0 (predose) to time τ, where τ = 12 hours; AUC_0-24_, area under the urine concentration-time curve over time 0 (predose) to 24 hours after dosing; AUC_0-48_, area under the urine concentration-time curve over time 0 (predose) to 48 hours after dosing; Ae total, total unchanged drug excreted in the urine; CLr, renal clearance; fe%, percentage of drug excreted; PK, pharmacokinetic.  Values are presented as geometric mean (%CVb). Gepotidacin PK data for Japanese participants is from the fed 1500 mg single dose and fed 2 × 3000 mg doses given 12 hours apart from the data presented herein. Gepotidacin PK data for Western participants are from healthy adult participants who participated in other cohorts of the overall study design and who underwent the same PK procedures as the Japanese participants; for the 2 × 3000 mg doses given 12 hours apart, dose 1 was gepotidacin administered alone and dose 2 was gepotidacin co-administered with digoxin and midazolam for a drug-drug interaction evaluation, with gepotidacin as the potential perpetrator. Refer to the respective main manuscript table for Western participant demographics.  ^a^ n = 10 for 2 × 3000 mg doses in Japanese participants.  ^b^ AUC_0-τ_ accounted for the first dose and fe% accounted for both doses when 2 doses were administered.  ^c^ n = 27 for the 1500 mg single dose in Western All Comers. | | | | | | | | | | |

**Figure S1.** Geometric mean and individual plasma AUC_0-t_ (A), AUC_0-∞_ (B), and C_max_ (C) of gepotidacin in Japanese participants after administration of a single 1500 mg dose (mesylate formulation) under fed and fasted conditions – mesylate formulation – to-be-marketed formulation study.


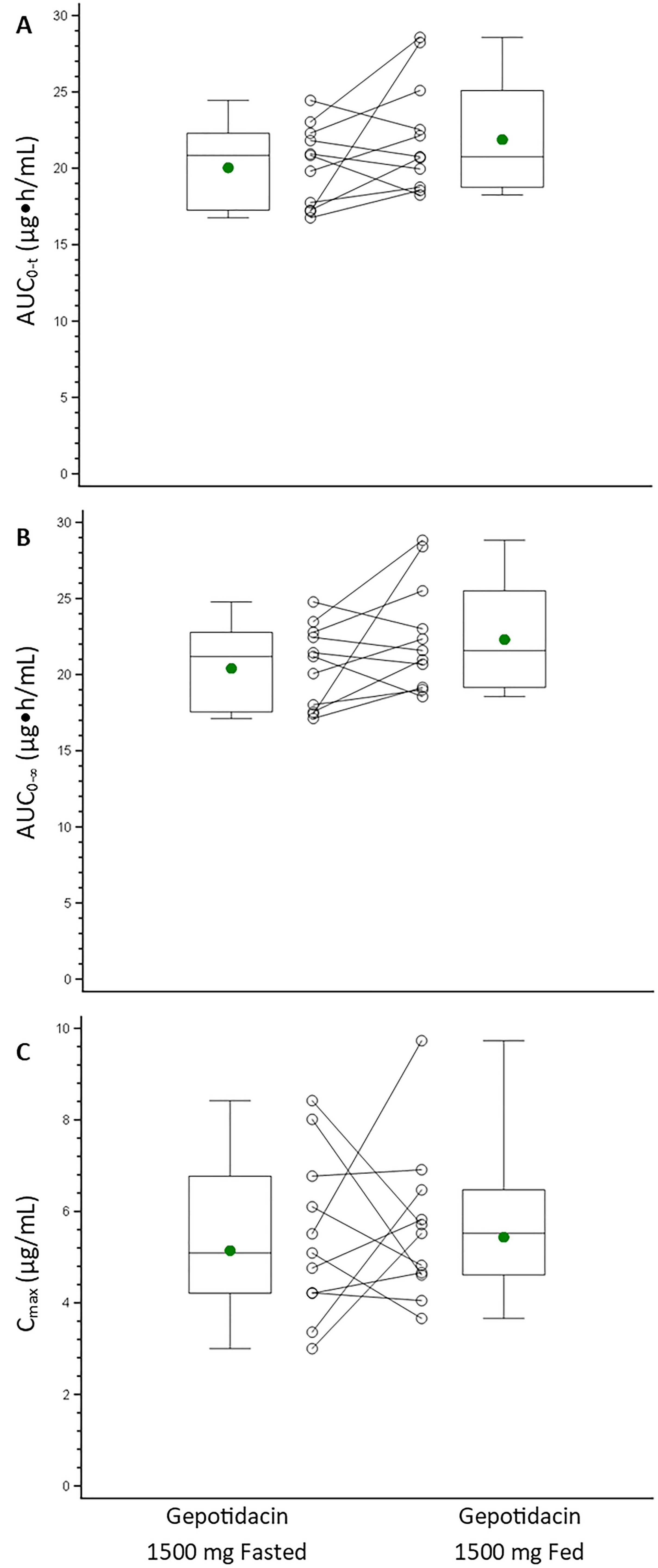

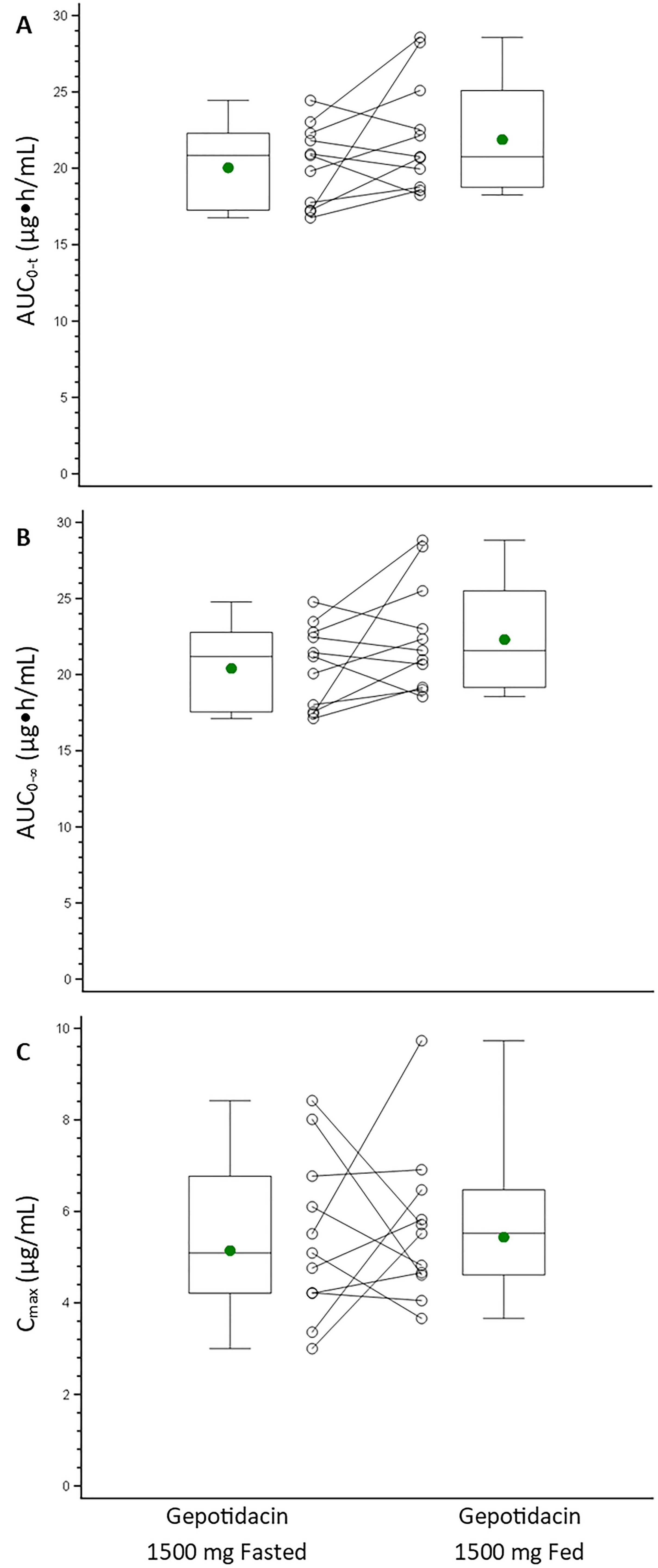

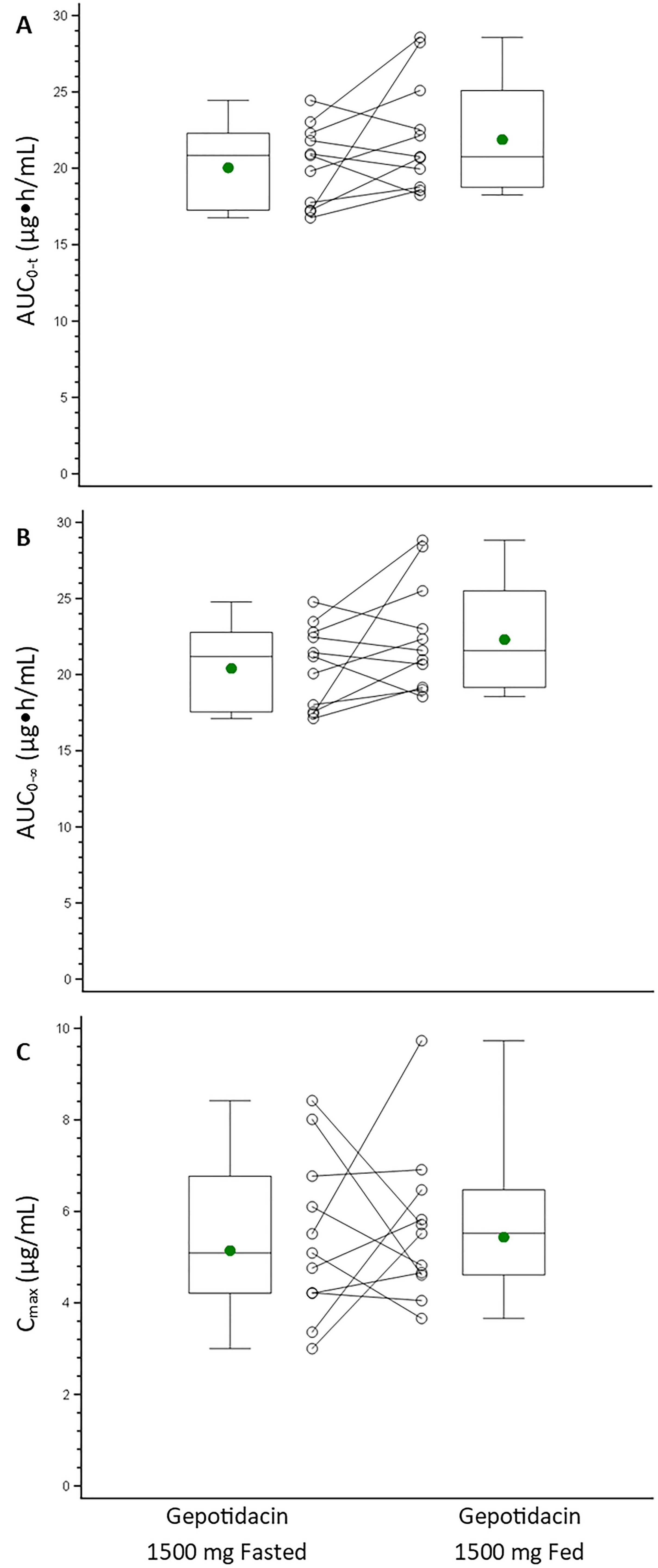


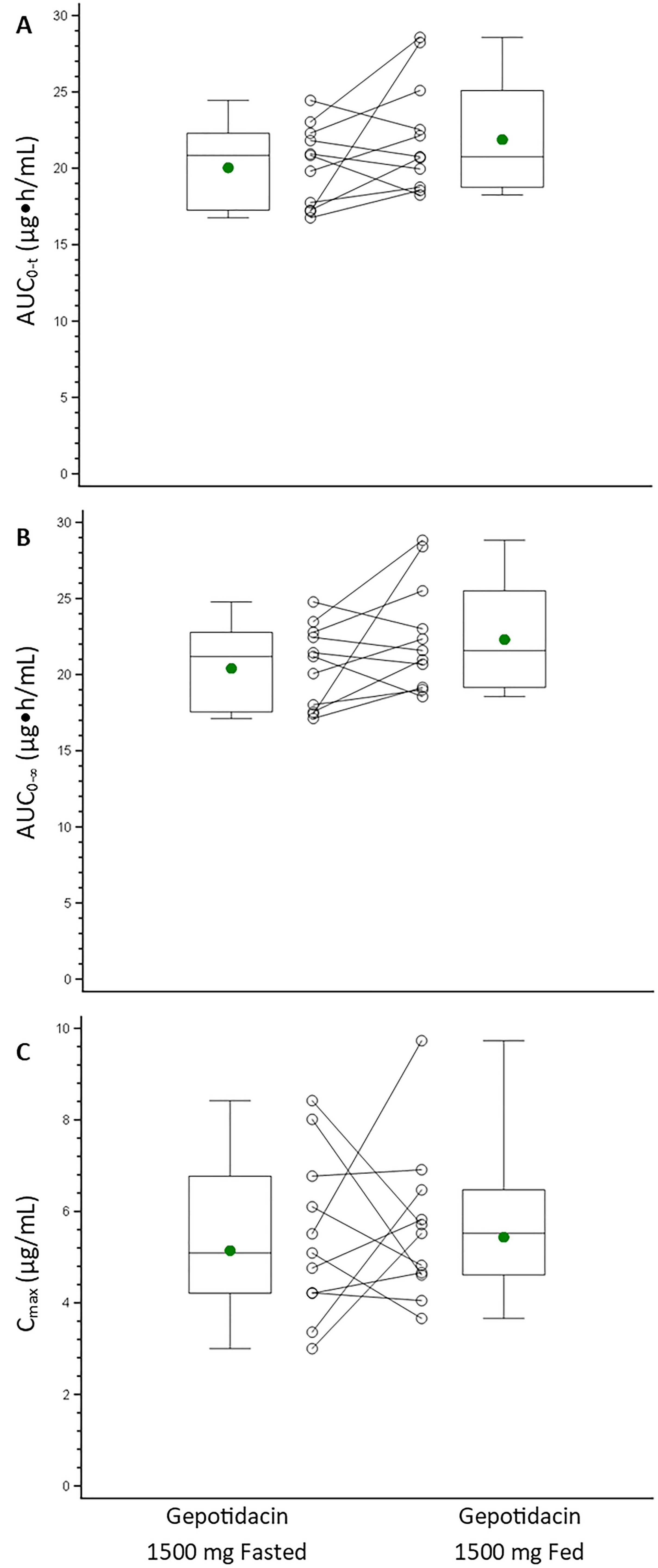

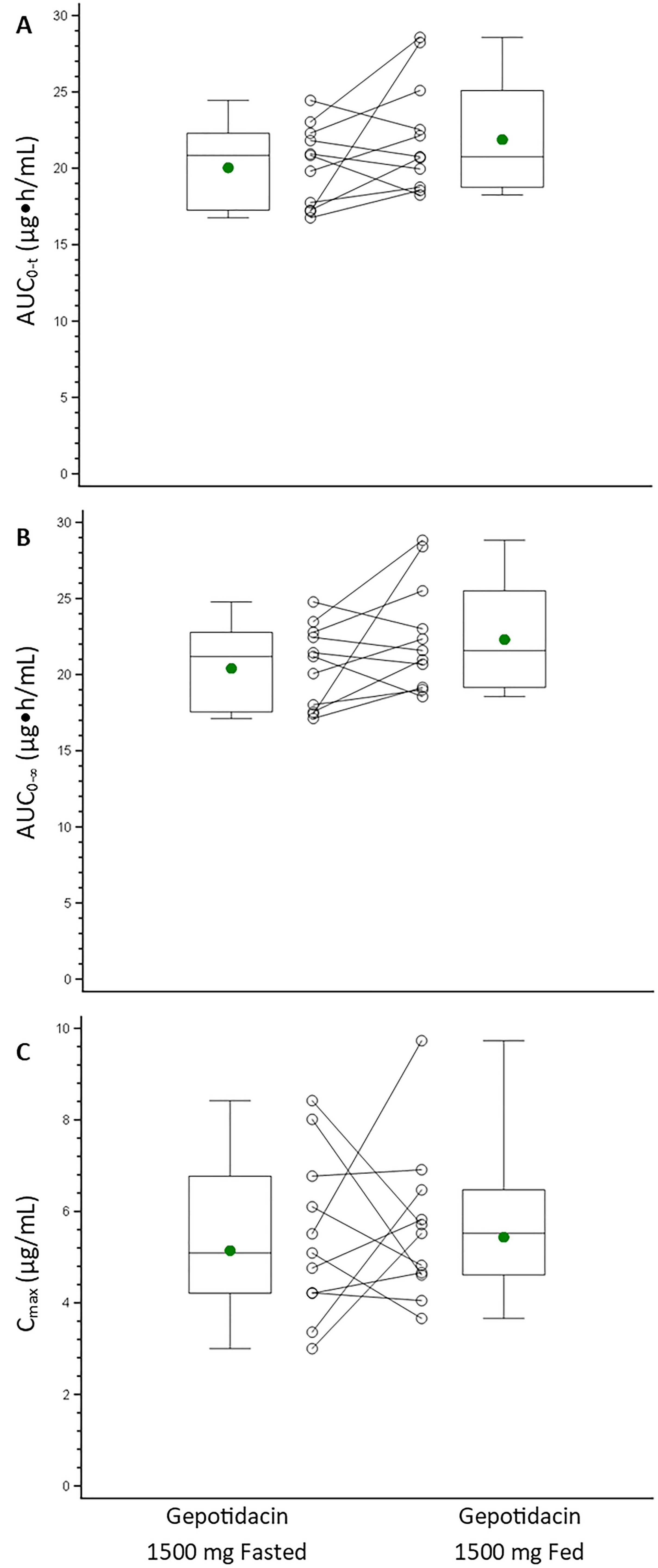

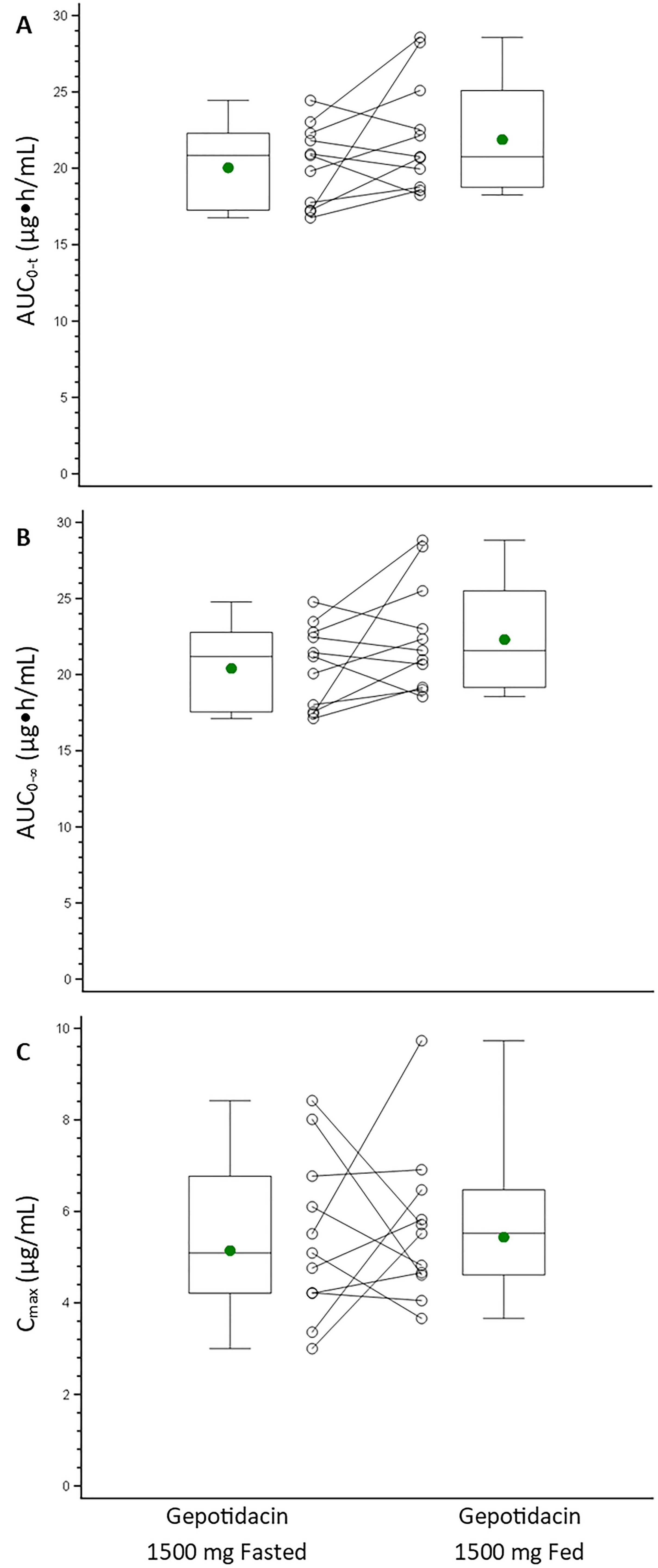


Bottom and top box lines represent the lower and upper quartiles, Q1 and Q3, respectively, with the median represented by the middle box line. Top and bottom whiskers represent the maximum and minimum values, respectively. Geometric mean is denoted by a closed green circle.
